# Short-Term Memory for Serial Order Moderates Aspects of Language Acquisition in Children With Developmental Language Disorder: Findings From the HelSLI Study

**DOI:** 10.3389/fpsyg.2021.608069

**Published:** 2021-04-20

**Authors:** Pekka Lahti-Nuuttila, Elisabet Service, Sini Smolander, Sari Kunnari, Eva Arkkila, Marja Laasonen

**Affiliations:** ^1^Department of Otorhinolaryngology and Phoniatrics, Head and Neck Surgery, Helsinki University Hospital and University of Helsinki, Helsinki, Finland; ^2^Department of Psychology and Logopedics, Faculty of Medicine, University of Helsinki, Helsinki, Finland; ^3^Department of Linguistics and Languages, Centre for Advanced Research in Experimental and Applied Linguistics, McMaster University, Hamilton, ON, Canada; ^4^Research Unit of Logopedics, University of Oulu, Oulu, Finland; ^5^Logopedics, School of Humanities, Philosophical Faculty, University of Eastern Finland, Joensuu, Finland

**Keywords:** non-verbal, serial short-term memory, developmental language disorder, specific language impairment, language acquisition

## Abstract

Previous studies of verbal short-term memory (STM) indicate that STM for serial order may be linked to language development and developmental language disorder (DLD). To clarify whether a domain-general mechanism is impaired in DLD, we studied the relations between age, non-verbal serial STM, and language competence (expressive language, receptive language, and language reasoning). We hypothesized that non-verbal serial STM differences between groups of children with DLD and typically developing (TD) children are linked to their language acquisition differences. Fifty-one children with DLD and sixty-six TD children participated as part of the HelSLI project in this cross-sectional study. The children were 4–6-year-old monolingual native Finnish speakers. They completed several tests of language and cognitive functioning, as well as new game-like tests of visual and auditory non-verbal serial STM. We used regression analyses to examine how serial STM moderates the effect of age on language. A non-verbal composite measure of serial visual and auditory STM moderated cross-sectional development of receptive language in the children with DLD. This moderation was not observed in the TD children. However, we found more rapid cross-sectional development of non-verbal serial STM in the TD children than in the children with DLD. The results suggest that children with DLD may be more likely to have compromised general serial STM processing and that superior non-verbal serial STM may be associated with better language acquisition in children with DLD.

## Introduction

The current study investigates an order processing mechanism that is assumed to contribute to short-term memory (STM) for both verbal and non-verbal sequences. We specifically explore the possible association of performance in non-verbal serial STM tasks to successful language acquisition. To this end, we introduce auditory and visual versions of a non-linguistic order matching task and report regression analyses of moderation effects on age-related improvement in measures of receptive and expressive language, as well as language reasoning, in children with typical and atypical language development.

Developmental language disorder (DLD) is currently proposed as a diagnostic label for children who have language problems that endure throughout their childhood and impact their everyday life but are not part of an identified biomedical condition, such as sensory-neural hearing deficit, neurological damage, or intellectual disability (Bishop et al., 2017). An older diagnostic label, specific language impairment (SLI), was used to characterize specifically delayed or disordered development of language in the presence of normal-range non-verbal abilities. A discrepancy between non-verbal and verbal ability was thought to be an expression of SLI. However, there is contemporary agreement acknowledging that children with DLD can also have deficits in their non-verbal abilities (Bishop et al., 2017) such as sustained attention (Finneran et al., 2009; Ebert and Kohnert, 2011), processing speed (Leonard et al., 2007), procedural learning (Ullman and Pierpont, 2005; Ullman et al., 2020), and working memory (WM) and STM (Leonard et al., 2007; Montgomery et al., 2010; Vugs et al., 2013; Archibald, 2017; Henry and Botting, 2017). The current study aims to investigate serial order processing as a necessary component in both non-verbal STM tasks and language acquisition, and to determine if it plays a role in DLD.

The possibility of a domain-general order processing mechanism contributing to STM tasks in different domains (e.g., verbal vs. visual and spatial) remains controversial in the literature (Hurlstone et al., 2014). Language unfolds in time, and the leading explanations of STM for representing the order in verbal lists are linked to assuming a context signal that changes with time over list positions (Burgess and Hitch, 1999). Recent work comparing serial memory of different materials has suggested that encoding linear order in time is carried out similarly for verbal and non-verbal material (Hurlstone and Hitch, 2015, 2018; Hurlstone, 2019). However, when the material is presented in temporal groups separated by longer pauses, a difference between domains is revealed. The order of verbal items is represented at two levels, at the whole-list level and at a separate within-temporal-group level, whereas the order of spatial and visual items is represented at the whole-list level only (Hurlstone and Hitch, 2015, 2018; Hurlstone, 2019). A moderate view is that memory for serial order in verbal, visual and spatial sequences has fundamental functional similarities (Hurlstone et al., 2014). Accordingly, it is coded largely in a domain-general fashion (Ginsburg et al., 2017), while it is possible that phonological STM also has a mechanism for domain-specific order processing, which is further separate from processing and storage of item information (Hartley et al., 2016; Majerus, 2019). Although disagreeing in some specifics (e.g., how the position in a sequence is coded), most serial STM models assume that item and serial order information are processed at least partly independently (although see Farrell and Lewandowsky, 2002, 2004). The current study investigates the possibility of a domain-general mechanism for representing the order in STM and hypothesizes a role for it in language development. Dysfunction of such a mechanism could affect the representation of order at the phonological level (phonemes, syllables) and at the higher levels (morphemes, words, phrases) of language. Consequently, performance in verbal STM tasks would be poor and learning of language chunks at different levels could be slowed down. Thus, weaker STM for order could link to DLD.

WM, the ability to maintain information in an accessible state for the performance of various tasks, has been hypothesized to be associated with DLD and other developmental cognitive disorders. In particular, the ability to temporarily bind together and rehearse verbal material in a speech-based code in WM, that is, functioning of phonological short-term memory (pSTM), *the phonological loop* component in the Baddeley and Hitch WM framework (Baddeley and Hitch, 1974; Baddeley, 2003), has been linked to individual differences in both typical and atypical language development. Poor pSTM in DLD has been reported in many studies (Gathercole and Baddeley, 1990, 1993; Baddeley, 2003; Archibald and Gathercole, 2006a; Montgomery et al., 2010; Verhagen and Leseman, 2016; Archibald, 2017). Early studies suggested that DLD may result from a deficit in the phonological storage component of the phonological loop (Baddeley et al., 1998; Archibald and Gathercole, 2006a,b). Attention-based WM (the *central executive* component in the Baddeley framework) is also assumed necessary for different aspects of language. Both pSTM and the central executive have frequently been found to be associated with lexical knowledge (Gathercole, 2006; Archibald, 2017), as well as syntactic knowledge (Marton and Schwartz, 2003) and sentence processing (Montgomery and Evans, 2009; Montgomery et al., 2010). The largest body of evidence and most theory-driven studies pertain to word learning (Gathercole, 2006). Gathercole (2006) has suggested that pSTM is especially critical in the early stages of acquiring vocabulary and that a phonological storage deficit can be detrimental to building a lexical knowledge base in long-term memory (LTM).

In the last 10 years, also non-verbal WM and STM have been reported to play a part in the atypical language development in DLD. Vugs et al. (2013) compared two hypotheses about the relationship between WM and DLD. The first is the phonological storage deficit hypothesis of DLD presented above (Gathercole and Baddeley, 1990; Baddeley et al., 1998; Archibald and Gathercole, 2006a,b). An alternative domain-general hypothesis of DLD asserted that non-verbal factors, including visuospatial storage capacity and attention-based executive functions in WM, have an additional role in the development of DLD (Vugs et al., 2013). In a meta-analytic study, Vugs et al. (2013) found support for the latter hypothesis. A further perspective is provided by the possibility that WM functions are underpinned by more primitive processing components, either domain-specific or domain-general. In the current study, we address the possibility that a domain-general mechanism for representing temporal order in STM may underlie individual performance differences in some, although not all, STM and WM tasks and thus could be linked to language development.

Verbal STM for item and serial order have been shown to be independently related to vocabulary acquisition in typical development (Majerus et al., 2006a,b; Leclercq and Majerus, 2010; Ordonez Magro et al., 2018). A recent study of typically developing (TD) 4–6-year-old children (Attout et al., 2020) found verbal serial order STM to be robustly linked to both receptive vocabulary, tested by picture matching, and expressive vocabulary, probed by picture naming. Better performance in a task with serial order reconstruction responses was also found to be related to faster novel word learning by 6–7-year-old TD children in experimental settings (Majerus and Boukebza, 2013). In a longitudinal study, Leclercq and Majerus (2010) found that verbal serial STM at the age of 4 years predicted receptive vocabulary 1 year later. In this study, memory for serial order was also linked to non-verbal reasoning. Therefore, serial processing capacity need not be domain-specific. It could also depend on a domain-general process.

There are only a few studies of DLD and STM for order. One study that did include children with DLD is that of Cowan et al. (2017) although its main target group was children with dyslexia. The researchers studied a large number of 7–9-year-old children performing both verbal and non-verbal serial STM tasks. Because of high comorbidity, the children with developmental dyslexia were divided into a group with DLD and another group without DLD. There were too few pure DLD cases to form a third atypical group. Both dyslexic groups were compared with TD children with typical reading and language. Cowan et al. found serial order memory deficits in both groups of children with dyslexia. The differences were especially robust in those serial memory tasks that made the use of mnemonic strategies difficult. Non-verbal intelligence also distinguished between the three groups: TD > dyslexic > dyslexic with DLD. When non-verbal intelligence was matched, both dyslexic groups had poorer serial STM than the TD group, but did not differ from each other. Although effects were clearer for verbal stimuli (digits) than non-verbal stimuli (shapes, spatial locations), Cowan et al. (2017) concluded a domain-general serial order memory deficit to be substantially related to dyslexia. This suggests that atypical serial STM is associated to DLD at least in those children that develop dyslexia in school age.

As reviewed above, STM for verbal serial order has been linked with language development (Majerus et al., 2006b; Majerus and Boukebza, 2013). This has been modeled in terms of the need to represent phoneme order in vocabulary learning and word order in acquisition of syntax (Gupta, 2003, 2006; Gupta and Tisdale, 2009). However, whether a domain-general order mechanism affects language acquisition in DLD is not presently known. In the present study, we asked whether domain-general serial STM is associated with language development in 4–6-year-old TD children and children with DLD. For this purpose, we developed two non-verbal order STM tasks: one with visual, the other with auditory stimuli. These tasks should be minimally confounded with existing language skills.

In the study of cognition, both domain specificity (e.g., verbal vs. non-verbal content) and sensory-modality specificity (e.g., visual vs. auditory vs. tactile) of serial order processing have been studied in the context of statistical learning (SL). SL refers to implicit ways of detecting and learning patterns and regularities in input. In the most commonly studied paradigms, stimuli (e.g., consonant-vowel syllables) are presented sequentially over time. Some sequences recur, whereas other sequences do not. The only cue to which sequences form recurring “words” are the transitional probabilities between the individual stimuli (e.g., the syllables). The original work by Saffran et al. (1996) presented auditory synthesized syllables. Later work has employed both verbal and non-verbal stimuli, presented visually, auditorily, or through touch. Both similarities and differences between SL of different stimulus classes have been found. In early comparisons, an artificial finite-state grammar describing recurring sequences of auditory tones was easier to learn than one describing vibro-tactile pulse sequences to fingertips or one describing the order of horizontal positions of black squares (Conway and Christiansen, 2005). In each case, the stimuli were drawn from a pool of five alternatives. However, later research showed that the relative difficulty of different sensory modalities interacted with the rate of presentation (Emberson et al., 2011).

There is no absolute consensus about the mechanisms that underlie SL. One main class of explanations of serial SL is based on an incremental acquisition of the transitional probabilities between the stimuli in the sequence. The other main class of explanations is based on the idea of sampling chunks of the sequence (for a review, see Perruchet, 2019). The two classes of explanation can also co-exist. Although SL is generally thought of as an implicit process, not available to conscious reflection, its results are usually tested by explicit forced-choice decisions. Recently, SL has also been shown to affect the accuracy of serial short-term memory tasks: better serial recall was seen for sequences that had first been included in an SL task (Isbilen et al., 2020). As SL has been shown to correlate with developmental language measures (for a review, see Siegelman, 2020) and has been suggested to play a role in DLD (Mainela-Arnold and Evans, 2014), possible modality-specific differences in the learnability of regularities in temporal SL sequences could also affect STM for serial order. From chunking explanations follows also an alternative hypothesis, i.e., that serial binding capacity reflected in STM tasks could constrain the ability of the SL mechanism to chunk the stream of incoming stimuli to build units in LTM. The effect of sensory modality on immediate serial memory was directly studied by Laasonen et al. (2012). Adult participants with or without dyslexia had to tell whether the stimulus order in two sequences was identical or not. The sequences were binary, i.e., they always consisted of only two kinds of stimuli. In the visual sequences, these were flashes of two spatially separated LED lights, in the auditory sequences, two tones of different pitch, and in the tactile sequences, stimulation of forefinger or middle finger. The mixed sequences included stimuli from two different modalities. With the same stimulus onset asynchrony in each modality and modality combination, the effect of the group was significant, but that of the modality did not approach significance. At least this serial STM task was not sensitive to sensory modality.

In the present study, we conceptualize domain-generality similarly as Endress (2019), who has proposed primitive operations that are duplicated within different domain-specific systems to be domain-bound rather than belonging to one domain-general module. The primitive processing component that we assume to be duplicated and bound to different cognitive systems involves the ability to represent order in time. We hypothesize that individual differences in this ability are reflected in serial STM for temporal sequences and in the efficiency of language acquisition. Because our hypothesis concerns a primitive component, we assume that it functions similarly in different sensory modalities. However, as in SL, we expect other aspects of, for instance, visual and auditory stimuli, and task details to contribute to the difficulty of serial STM tasks, making them each unique despite a common serial component.

Here we hypothesized that children with DLD have poorer serial STM capacity than TD children. Furthermore, we hypothesized that a domain-bound capacity for serial order plays a role in language acquisition so that the development of serial STM capacity moderates improvement with age in language competence. We explored non-verbal serial STM moderation effects to three aspects of language competence (expressive and receptive language and language reasoning) and hypothesized that the moderation effect is found for all three. If the relationship between a general serial STM impairment and DLD exists, assessing general serial STM when DLD is suspected could be helpful, especially if the verbal assessment is challenging, as it is, for example, with bilingual children who have poor L2 skills.

## Materials and Methods

### Participants

There were 51 children (39 boys) with DLD and 66 TD children (52 boys) between the ages of 4 and 6 years (Mean = 5 years 5 months, sd = 10.2 months). All children were taking part in the more extensive HelSLI Study (Laasonen et al., 2018, see also the Acknowledgments section) and were native monolingual Finnish speakers with no gross neurological findings. Based on caregiver reports, the hearing of the children was normal. In Finland, hearing is screened for all newborn babies and actively followed until school-age. The children with DLD were required to have a performance intelligence quotient (PIQ) of at least 70. Parental consent was obtained for each child. Ethical approval for the project was granted by the ethical board of the Helsinki Uusimaa Hospital District.

The children with DLD were part of the clientele of the Audiophoniatric Ward for Children in the Department of Phoniatrics of Helsinki University Hospital. They had been referred to the ward because of suspected DLD. They were examined and assessed during their visits to the ward. Diagnoses of other developmental disorders were used to exclude some potential participants. The children with DLD included in this study were all diagnosed using ICD-10 classification codes as having F80.1 (expressive language disorder), F80.2 (receptive language disorder), F80.8 (other developmental disorders of speech and language), or F83 (a mixture of specific developmental disorders including speech and language), and, not having a hearing impairment, intellectual disability, autism spectrum disorder, oral anomalies, or a diagnosed neurological impairment or disability (e.g., epilepsy, chromosomal abnormalities). More precise inclusion/exclusion criteria can be found in an article describing the HelSLI research project (Laasonen et al., 2018).

The TD group consisted of volunteer children from kindergartens in the metropolitan area of Helsinki, with a PIQ of at least 85 and no diagnosed or suspected language difficulties, except for possible minor articulation impediments.

The different inclusion criteria with respect to PIQ ensured a large enough sample size in the DLD group. Originally, we intended to form a separate group for children with DLD and PIQ < 85. However, because only 15 children with DLD had a PIQ between 70 and 84, this would have led to too small a sample size to warrant a separate group. Our exclusion criteria for the children with DLD were otherwise rigorous (Laasonen et al., 2018). In the initial analyses of data, the children with DLD with PIQ between 70 and 84 were not found to qualitatively differ from the children with DLD that had PIQ scores of 85 and above, with regard to the relationships between the variables analyzed in this study. It is not likely that this inclusion criterion difference altered the observed results except by adding statistical power, as sample size and PIQ variance increased. However, as a control measure, non-verbal reasoning was statistically controlled in the analyses.

Descriptive statistics of both groups are presented in Table 1, and distributions of the main variables are shown in Supplementary Figure 1. Because of the clinical context and the participants’ young age, some language and cognitive tests ended up with missing values, for instance, if a child refused to co-operate on a task. Frequencies of these are also reported in Table 1. As a group, the TD children were slightly older than the children with DLD. This was an unforeseen result of delays in the data collection and the clinical setting (the children with DLD being studied more likely near their birthdays). However, age was also used as a regressor variable. The considerable overlap of age distributions justified studying the age interaction effects that were our primary interest. Only at either end of the age distribution must caution be applied.

**Table 1:** Age and test scores: Means, standard deviations, missingness, and ranges of TD children and children with DLD, and effect sizes of mean comparisons.

**Variable**	**Group**						***d***^**a**^
	**TD (*****n*** **=** **66)**			**DLD (*****n*** **=** **51)**			
	**M (SD)**	**N of missing**	**Range**	**M (SD)**	**N of missing**	**Range**	
Age (months)	67.0 (10.0)	–	50–86	63.1 (10.2)	–	49–82	0.39^†^
Non-verbal Reasoning^b^^,^^c^	0.3 (0.8)	–	(−1.1)−2.2	−0.4 (0.9)	–	(−2.2)−2.1	0.92^***^
Matrix Reas., Raw Score^b^	17.6 (4.2)	–	8–26	13.4 (4.7)	–	4–26	0.94^***^
Matrix Reas., Std. Score^b^	11.6 (2.6)	–	5–17	9.3 (2.4)	–	3–16	0.88^***^
Block Design, Raw Score^b^	28.2 (4.2)	–	20–38	25.0 (4.1)	–	18–36	0.75^***^
Block Design, Std. Score^b^	10.5 (2.8)	–	4–16	8.6 (2.7)	–	4–16	0.67^***^
**Expressive language variables**							
BNT, Raw Score	33.4 (7.1)	1	16–52	15.1 (9.1)	9	0–32	2.10^***^
RDLS Expr., Raw Score	41.5 (8.4)	–	20–54	20.7 (12.0)	12	0–42	1.86^***^
EOWPVT, Raw Score	83.9 (16.5)	1	54–120	44.8 (23.1)	5	0–79	1.95^***^
Pict. Naming, Raw Score^b^	23.3 (2.8)	–	17–29	13.4 (6.6)	–	0–25	2.06^***^
Pict. Naming, Std. Score^b^	10.5 (3.0)	–	2–17	3.3 (3.0)	–	1–12	2.42^***^
**Receptive language variables**							
Receptive Voc., Raw Score^b^	31.6 (2.5)	2	24–36	25.9 (5.7)	–	9–35	1.37^***^
Receptive Voc., Std. Score^b^	10.9 (2.2)	2	5–15	6.5 (3.9)	–	1–15	1.44^***^
RDLS Compr., Raw Score	55.9 (3.8)	–	46–62	47.0 (9.1)	5	26–59	1.31^***^
ROWPVT, Raw Score	112.9 (34.2)	–	58–178	65.0 (24.3)	6	33–143	1.51^***^
**Complex language reasoning variables**							
Vocab., Raw Score^b^	27.8 (10.5)	–	5–48	9.5 (6.1)	–	0–22	2.08^***^
Vocab., Std. Score^b^	10.7 (3.0)	–	2–18	4.8 (1.7)	–	1–8	2.31^***^
Inform., Raw Score^b^	27.2 (3.1)	–	20–33	17.7 (5.9)	–	0–28	2.08^***^
Inform., Std. Score^b^	10.8 (2.5)	–	4–17	3.1 (2.4)	–	1–9	3.11^***^
Word Reas., Raw Score^b^	21.3 (3.5)	–	10–28	7.3 (7.5)	–	0–23	2.50^***^
Word Reas., Std. Score^b^	10.3 (2.4)	–	2–16	2.4 (2.5)	–	1–10	3.20^***^
Compr. Instr., Raw Score	20.9 (3.4)	–	14–29	14.2 (5.0)	–	4–26	1.60^***^
Compr. Instr., Std. Score	9.2 (2.4)	–	3–15	5.2 (2.6)	–	1–12	1.61^***^
**Variables for non–verbal STM validation**							
Sentence Repet., Raw Score	21.0(3.5)	1	12–28	8.5 (5.9)	1	0–23	2.68^***^
Forward Mem., Raw Score	14.8 (3.5)	–	4–22	8.1 (4.9)	2	0–19	1.63^***^

*TD, Typically developing children; DLD, Children with developmental language disorder; n, Number of observations; M, Mean; SD, Standard deviation; d, Cohen’s d, effect size; Matrix Reas., Matrix Reasoning; Vocab., Vocabulary; Inform., Information; Word Reas., Word Reasoning; Compr. Instr., Comprehension of Instructions subtest from the Nepsy–II; BNT, Boston Naming Test; RDLS, Reynell Developmental Language Scales III; Expr., Expressive Scale; Compr., Comprehension Scale; EOWPVT, Expressive One Word Picture Vocabulary Test; Pict. Naming, Picture Naming; Receptive Voc., Receptive Vocabulary; ROWPVT, Receptive One Word Picture Vocabulary Test; Sentence Repet., Sentence Repetition subtest from the NEPSY-II; Forward Mem., Forward Memory subtest from Leiter International Performance Scale—Revised; Std. Score, Standard score*.

a *In the case of missing values, d and p-values are pooled from the independent samples t-tests in twenty multiple imputations*.

b *Wechsler Preschool and Primary Scale of Intelligence, Third edition (Wechsler, 2009)*.

c *Non-verbal reasoning score is the mean of sample standardized z scores of Matrix reasoning and Block design raw scores*.

† *p = 0.034*,

*** *p < 0.001*.

### Language and Cognitive Tests

The selection of psychometric instruments was restricted to those available in Finnish. All presented tests were Finnish versions. We used the subtests Picture Naming, Receptive Vocabulary, Information, Vocabulary, Word Reasoning, Block Design, and Matrix Reasoning from the Wechsler Preschool and Primary Scale of Intelligence—Third Edition (WPPSI-III) (Wechsler, 2009). The Comprehension of Instructions and the Sentence Repetition subtests came from the Nepsy-II (Korkman et al., 2008), the Forward Memory from the Leiter International Performance Scale—Revised (Roid and Miller, 1997), and the Comprehension and Expressive Scales from Reynell Developmental Language Scales III (Edwards et al., 1997) (RDLS-III). We also employed the Expressive (EOWPVT) and Receptive (ROWPVT) One-Word Picture Vocabulary Tests (Martin and Brownell, 2010, 2011) as well as the Boston Naming Test (BNT) (Kaplan et al., 1983). Appendix 1 has a short description of each test. We used the raw scores of these variables, their sample-centered transformations, and sample-standardized z-transformations.

Because the full PIQ may be sensitive to language competence in young children, we used the mean of sample-standardized z scores of Matrix reasoning and Block design raw scores as an index of non-verbal reasoning. The TD group had a higher mean in non-verbal reasoning than the group with DLD nonetheless. This difference is in line with the one found by Gallinat and Spaulding (2014) in their meta-analysis and was also expected because extensive matching of groups was not feasible (Laasonen et al., 2018). Accordingly, we statistically controlled for the non-verbal reasoning composite, including it as a covariate in the analyses.

### Non-verbal Serial STM Tasks

**Figure 1:**
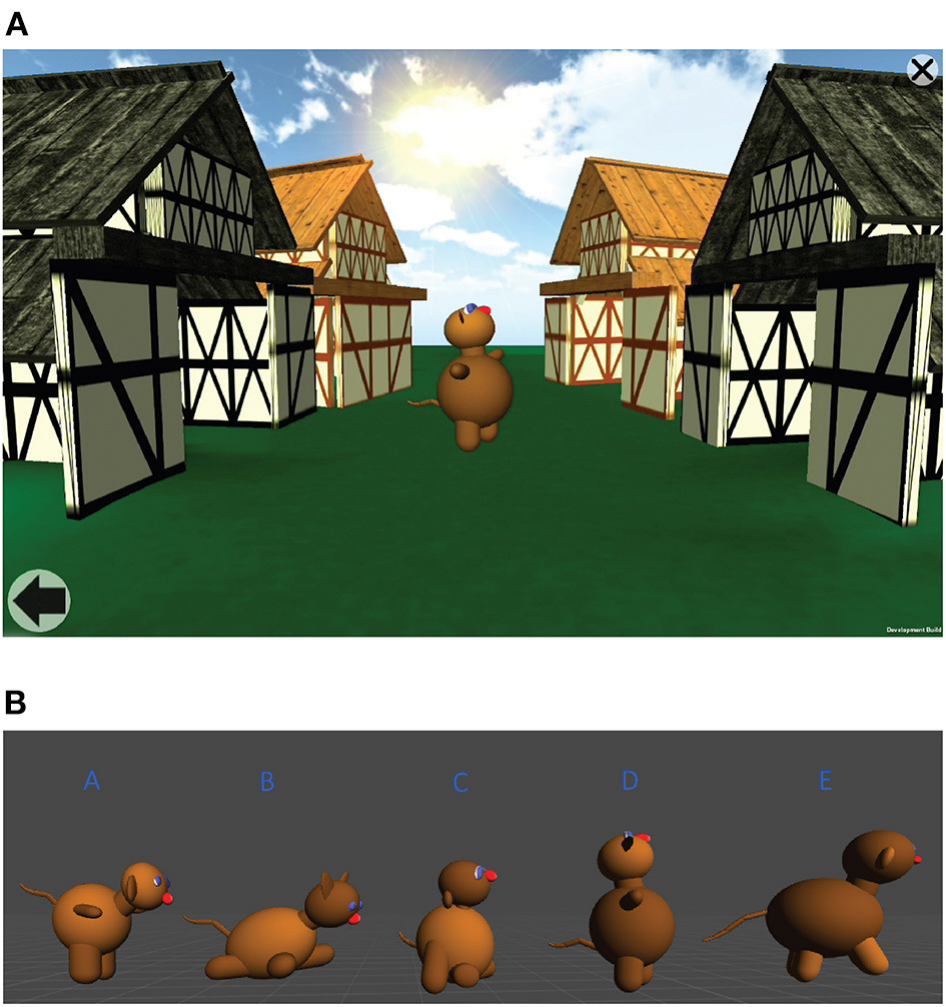
**(A)** Screen capture from the visual serial STM task. **(B)** Animal figures used in visual serial STM task.

Matching tasks involving pairs of sequences in the visual and auditory modalities were created for tablet computers with touch screens. Both the visual and auditory STM tasks were non-verbal. In each of them, lengthening pairs of stimulus sequences involving animated fantasy animals or reverse-played animal sounds were presented for order comparison (see Figure 1; a short demonstration video is also available as Supplementary Material). The child’s task was to tell if the order of the stimuli in the two sequences was the same or not.

In the visual task, two pairs of barns facing each other were pictured on the screen. Sequences of fantasy animals moved from left to right between the two upper barns (first sequence) and the two lower barns (second sequence), respectively. A pool of five different fantasy animals was used as stimuli. All the animal figures were constructed from the same 13 basic shapes of similar coloring; only the body parts’ proportions and positioning varied. Each animal also had a distinctive movement pattern. To minimize demands on item memory, we presented only binary sequences, such that each sequence consisted of tokens of only two different animal shapes, which had been sampled from the pool of five possible stimuli. The animals moved along constant horizontal paths in the central part of the screen. The first sequence of animals followed a path noticeably above the horizontal midline (between the upper barns, referred to as “Matt’s barns”), and the second sequence traveled below it (between the lower barns, “Mary’s barns”). Only one animal was seen at a time. Each animal was visible for ~1,500 ms completing its path between two barns. Thus, the participants had to bind the stimuli to a temporal sequence in their WM.

In the auditory task, we selected stimuli from five separate sound files consisting of animal calls played backward. Each call was ~1,500 ms long. During the auditory task, the same initial image of the two upper and two lower barns as in the visual task was now lightly dimmed to look dark on the screen. During the first sequence in a pair, the upper right-side barn was lit during each call and again dimmed after the call, signaling that animals in Matt’s barn said “good night.” During the second sequence in a pair, the lower right-side barn was lit similarly when Mary’s animals said “good night.”

The tasks were performed on a tablet computer—either Samsung Galaxy Tab 3 10.1 (2014) or Samsung Galaxy Tab A 10.1 (2016), running Android 5.0–7.0. Tab 3 had a 10.1-inch WXGA TFT display with a resolution of 1,280 × 800 pixels, while Tab A had a 10.1-inch TFT LCD screen with a resolution of 1,920 × 1,200 pixels. The STM tasks were custom-created applications based on the Unity engine (Unity Technologies). Each STM task—auditory and visual—was described to the child as a computer game (See Figure 1, Supplementary Movie Clip 1).

The “game” consisted of several rounds. In each round, six pairs of binary stimulus sequences of the same length were presented to the child. Two different stimuli were presented on each trial. After each trial, the child was asked to tell if the animals in the two sequences (Mary’s and Matt’s animals) had been in the same or a different order. In a round, there were always three trials in which the stimuli in the sequence pairs were in the same order and three in which the orders were different. Before the first round, children were presented with five practice trials. The practice trials were similar to the experimental trials (see below), with the first three consisting of sequences of two stimuli and the last two consisting of sequences with three stimuli. For the TD children, the instructions were given verbally, and for the children with DLD also symbol pictures were used when needed. During the practice trials, feedback was issued and, when needed, further instructions and practice were provided. The first experimental round of trials consisted of sequences of two stimuli. If the child responded correctly to four out of the first six comparisons, sequence length was increased with one more stimulus. When a child answered incorrectly on three or more trials on a round, the task was terminated. It took ~5–15 min for each child to complete the STM tasks.

The order of the stimuli in a sequence was pseudorandom, but the same for all children. In the SAME trials, both sequences were precisely the same, and in the DIFFERENT trials, two different consecutive stimuli had changed places in the second sequence. When the sequence length was four or more stimuli, the difference was always among n−2 middle stimuli, never including the first or last stimuli. The two sequences were presented one after the other with a 3-s inter-sequence interval. Within a sequence, the stimulus onset asynchrony was 2 s.

The presentation order was balanced between the visual and auditory tasks: half of the children performed the auditory task first and half the visual task first. A child was instructed to judge whether two stimulus sequences were similar or not. The child responded by touching either a virtual green button with a black ✓-symbol (SAME) or a virtual red button with a black × -symbol (DIFFERENT) on the tablet screen. The virtual buttons had a diameter of 30 mm, “SAME” on the left side of the touchscreen and “DIFFERENT” symmetrically on the right side.

We first computed separate STM scores for the auditory and visual tasks: the sum of the correct answers of all trials that had been presented. If a child had answered correctly on the first four trials on a round, the last two trials were not presented to shorten the testing time but were credited as correct. This scoring is similar as in, for instance, Archibald and Gathercole (2006b). Both visual and auditory STM task scores were standardized, and a composite STM score was computed as an average. Descriptive statistics for both groups are presented in Table 2, and distribution of the composite variable is shown in Supplementary Figure 1.

**Table 2:** Serial STM tasks and language composite scores: Means, standard deviations and ranges of TD children and children with DLD, and effect sizes of mean comparisons.

**Variable**	**Group**				***d***^**a**^
	**TD (*****n*** **=** **66)**		**DLD (*****n*** **=** **51)**		
	**M (SD)**	**Range**	**M (SD)**	**Range**	
**Serial short-term memory**					
Visual serial STM	7.3 (7.2)	1–30	5.3 (4.6)	1–21	0.32^*†*^
Auditory serial STM^b^	10.2 (8.4)	1–35	6.2 (5.3)	1–24	0.55^*††*^
Serial STM composite	0.2 (0.9)	(−0.8)−2.8	−0.2 (0.6)	(−0.8)−1.6	0.52^*†††*^
**Language**					
Expressive language composite	0.6 (0.5)	(−0.5)−1.5	−0.8 (0.8)	(−2.5)−0.6	2.25^***^
Receptive language composite	0.5 (0.5)	(−0.7)−1.4	−0.7 (0.9)	(−2.4)−1.1	1.70^***^
Complex language reasoning composite	0.6 (0.5)	(−0.4)−1.4	−0.8 (0.7)	(−2.0)−0.8	2.48^***^

*TD, Typically developing children; DLD, Children with developmental language disorder; n, Number of observations; M, Mean; SD, Standard deviation; d, Cohen’s d, effect size; STM, Short term memory*.

a *For visual serial STM d and p-values are from original data since it was the only one of these variables without any missing values. For all other variables d and p-values are pooled from the independent samples t-tests in twenty multiple imputations*.

b *One TD child had auditory serial STM task missing*.

† *p = 0.074*,

†† *p = 0.003*,

††† *p = 0.005*,

*** *p < 0.001*.

Since our interest was in a domain-general aspect of serial STM, and, to reduce the effect of random variability and task-specific strategies, we used a composite score instead of using visual and auditory scores separately. Previous findings of Laasonen et al. (2012) suggest that processing of order in visual and auditory unimodal and crossmodal temporal sequences with stimuli of similar complexity might be a modality-general capacity.

### Overall Procedure

The cognitive and language performance of children with DLD was tested in a clinical examination by neuropsychologists and speech-language pathologists during the children’s visits to the ward. The STM tasks reported here were presented at convenient times in their assessment schedule. The TD children completed the same speech and language and neuropsychological assessment batteries (for details, see Laasonen et al., 2018). The TD children were assessed in a quiet room in their kindergarten. For 15 children with DLD and 50 TD children, the auditory and visual STM tasks were presented in different sessions and on different days, but mostly within 6 days of each other (for one TD child within 8 days and for another within 11 days).

### Statistical Analysis

Based on the 11 tests targeting language functions, we investigated receptive and expressive language factors and a factor representing more complex language reasoning functions. This structure was outlined a priori and tested with confirmatory factor analysis (CFA) for the raw scores of the 11 language tests. CFA was carried out with MPlus 8 (Muthén and Muthén, 1998–2017) using the MLMV estimator^1^ on the total sample of children. For the 11 observed language variables, we hypothesized a hierarchical factor model with three factors (receptive language, expressive language, and language reasoning) at the first level and a higher-order general language factor explaining factor correlations. Based on the CFA structure, three language composites were formed as an average of sample standardized values of the respective observed variables. An expressive language composite was formed from WPPSI-III Picture Naming, EOWPVT, BNT, and the Expressive Scale from RDLS-III; a receptive language composite from WPPSI-III Receptive Vocabulary, ROWPVT, and Comprehension Scale from RDLS-III; and a language reasoning composite from WPPSI-III Information, Vocabulary and Word Reasoning subtests, and Nepsy-II Comprehension of Instructions.

The distributions of the STM tasks were positively skewed, and visual inspection of bivariate scatterplots and residuals indicated heteroscedasticity. Statistical tests confirmed this in the distributions. For this reason, we used linear regression analyses with heteroscedasticity-consistent (HC4) standard error estimators (Cribari-Neto and Zarkos, 2004; Hayes and Cai, 2007). For the variables with missing values, we used the multiple imputation procedure with twenty imputed datasets, fully conditional specification (chained equations) iterative Markov Chain Monte Carlo method with the constraints of zero minimum and observed non-missing sample maximum. Small-sample degrees of freedom (Reiter, 2007; Van Ginkel and Kroonenberg, 2014) were used, and here the pooled results are reported. The multiple imputation procedure was applied to the raw scores of the language, cognitive, and STM variables before centering or standardizing with gender, group status, and age also in the imputation model.

Our primary interest in the analyses lay with revealing moderator effects on language development by exploring STM × age × group -interactions. The explanatory variables were mean-centered for the estimation of unstandardized effects. We did this to make the interpretation of the estimates more comprehensible and practical. For centered variables, zeros correspond to the values of the original sample means instead of their original zero values (Hayes and Rockwood, 2017). To estimate standardized effects, the variables were standardized, and interaction effects were calculated as products of these. The GLM procedure of SPSS 25.0.0.2 was used for these analyses. The conditional effects were also cross-checked using the PROCESS macro (Hayes, 2018), and tests of conditional effects were estimated separately for each imputation sample. Again, the results from all 20 samples were pooled using small-sample degrees of freedom (Reiter, 2007; Van Ginkel and Kroonenberg, 2014). We used two-tailed statistical significance tests and set α = 0.05 for the omnibus effects, acknowledging that the combination of heteroscedasticity correction and small-sample degrees of freedom may result in an overly conservative testing procedure. The conditional effect of serial STM × age was estimated for each group if there was a statistically significant three-way interaction of serial STM × age × group.

## Results

### Confirmatory Factor Analysis of Language Variables

Among the 11 language variables opted for a CFA, there were a few unequal bivariate correlations for the two groups. The CFA was performed for the joint TD + DLD. The differences in performance level between the groups likely increased the covariances between the language factors somewhat. The correlations between all of the variables are in Supplementary Table 1. The CFA model is presented in Supplementary Figure 2.

The CFA on the 11 language variables had a good fit ($\chi_{43}^{2}=\;$77.9, *p* = 0.0009, RMSEA = 0.075, 90% confidence interval for RMSEA = [0.048, 0.102], CFI = 0.973, TLI = 0.965, SRMR = 0.029, BIC = 10351.8). Tests of receptive and expressive language loaded highly on their corresponding factors, and tests of more complex language functions loaded together on the language reasoning factor. This factor resembles the verbal intelligence quotient (VIQ), which is composed of three of the WPPSI-III variables loading on this factor.

All of the three language composites had high reliabilities in the total sample (alpha reliabilities 0.86–0.96, see Table 3). When estimated separately in TD children, the reliabilities were also high (for expressive language composite α = 0.87, for receptive α = 0.68, and for language reasoning α = 0.72) and, respectively, in children with DLD (for expressive α = 0.92, for receptive α = 0.80, and for language reasoning α = 0.89).

**Table 3:** Correlations of age and composite scores.

**Variable**		**TD** **DLD**							
		**1**.	**2**.	**3**.	**4**.	**5**.	**6**.	**7**.	**8**.
1. Age (months)	Total sample	n.a.	0.67^***^ 0.76^***^	0.50^***^ 0.41^***^	0.50^***^ 0.58^**^	0.51^***^ 0.66^***^	0.56^***^ 0.59^***^	0.76^***^ 0.68^***^	0.72^***^ 0.75^***^
2. Non-verbal Reasoning		0.71^***^	0.84	0.56^***^ 0.43^**^	0.50^***^ 0.58^***^	0.56^***^ 0.65^***^	0.50^***^ 0.52^***^	0.59^***^ 0.65^***^	0.46^***^ 0.71^***^
3. Serial STM composite		0.49^***^	0.54^***^	0.57^*a*^	0.37^**^ 0.34^*^	0.49^***^ 0.45^**^	0.36^**^ 0.16	0.39^**^ 0.30^*^	0.37^**^ 0.37^**^
4. Sentence Repetition^b^		0.47^***^	0.62^***^	0.39^***^	n.a.	0.39^**^ 0.53^***^	0.64^***^ 0.77^***^	0.62^***^ 0.52^***^	0.64^***^ 0.72^***^
5. Forward Memory^c^		0.56^***^	0.69^***^	0.49^***^	0.73^***^	n.a.	0.38^**^ 0.43^**^	0.43^***^ 0.57^***^	0.52^***^ 0.64^***^
6. Expressive language composite		0.51^***^	0.62^***^	0.34^***^	0.89^***^	0.68^***^	0.96	0.76^***^ 0.54^***^	0.71^***^ 0.75^***^
7. Receptive language composite		0.64^***^	0.69^***^	0.40^***^	0.77^***^	0.71^***^	0.79^***^	0.86	0.73^***^ 0.82^***^
8. Complex language reasoning composite		0.59^***^	0.67^***^	0.40^***^	0.89^***^	0.78^***^	0.89^***^	0.88^***^	0.93

*Total group correlations are below, and group-wise correlations are above the diagonal. Internal consistencies (Cronbach’s α) for the total group are underlined on the diagonal*.

*TD, Typically developing children; DLD, Children with developmental language disorder; n.a., Not applicable; STM, Short-term memory*.

a *If the one TD child that had the largest difference between visual and auditory serial STM tasks is left out, α = 0.63*.

b *Sentence Repetition is from the NEPSY-II*.

c *Forward Memory is from Leiter International Performance Scale—Revised*.

* *p < 0.05*,

** *p < 0.01*,

*** *p < 0.001 for the null hypothesis ρ = 0*.

### Non-verbal Serial STM Composite Validity and Reliability

TD children performed better than children with DLD in both serial STM tasks, although the difference in the visual task did not quite reach the preselected *p*-level. However, the effect size was moderate also in the visual task (see Table 2). The visual and auditory tasks correlated significantly in the total sample (*r* = 0.40, *p* < 0.001). The relationship between the two tasks remained statistically significant even when partialling out age and non-verbal reasoning (*r* = 0.24, *p* = 0.011). Separately, in the sample of TD children, the tasks correlated (*r* = 0.37, *p* < 0.01) and in the sample of children with DLD (*r* = 0.42, *p* < 0.01).

To validate that the composite qualifies as a measure of serial STM, we correlated both serial STM tasks and the serial STM composite with two viable STM tasks, i.e., Sentence Repetition and Leiter Forward Memory. The former represents verbal and the latter visuospatial STM. Correlations between the serial STM composite and the other STM tasks are presented in Table 3. These show moderate relationships and indicate the validity of the serial STM composite. The relationships remained moderate even when partialling out age (*r* = 0.21, *p* = 0.024 for Sentence Repetition and *r* = 0.30, *p* = 0.001 for Forward Memory). Partialling out also non-verbal reasoning reduced partial correlations (*r* = 0.07, *p* = 0.455 for Sentence Repetition and *r* = 0.17, *p* = 0.067 for Forward Memory).

The alpha reliability of the serial STM composite proved to be adequate. The fairly small number of trials in the serial STM tasks, dictated by practical constraints, allowed guesses a greater role in the number of correct answers than we had anticipated. If the one TD child who had the largest discrepancy between visual and auditory serial STM tasks were left out from the estimation of alpha, the coefficient would be 0.63, which can be considered acceptable. When estimated separately for TD children, the reliability was satisfactory, α = 0.61 and likewise for children with DLD, α = 0.60.

There were 20 children in both groups that did not produce four correct responses in the shortest sequence length of two in either or both of the STM tasks. We checked that this possible “floor effect” did not change the results of the moderation by running the analyses also without these children. The smaller sample sizes weakened statistical power, but the effects were comparable to those reported below.

### Correlations Between Age, Non-verbal Serial STM, and Language

Correlation coefficients between the main variables in the total sample and for the TD and DLD groups separately are presented in Table 3. The complete correlation matrix is available in Supplementary Table 1. Some correlations for the two groups appear different in strength indicating different relationships between these variables in TD and DLD groups. The differences were tested both for the matrices and for each of the 28 individual pairs of correlation coefficients. Box’s test was run for the correlation matrix of age, non-verbal reasoning, serial STM composite, and three language composites. It showed statistically significant differences in group correlations for the whole variable set (*F*_21,42436_ = 4.1, *p* < 0.001). However, when individual bivariate correlation coefficients were tested, there were no significant differences between the groups (smallest *p* = 0.100 for the correlation of non-verbal reasoning with the language reasoning composite, *r*_*TD*_ = 0.46 vs. *r*_*DLD*_ = 0.71). Seemingly different is the correlation between serial STM and expressive language composites which is not statistically significant in the DLD group. There were some more missing values in the expressive variables among the children with DLD (see Table 1). These arose from the children occasionally declining to take individual tests. It looks like the group difference was larger on expressive than on receptive tests, which suggests that perhaps children with DLD succeeded more poorly on them. As a consequence, the measurement of expressive language may be less valid than the measurement of receptive language.

As the multivariate relationships between the variables were not the same in the TD and DLD groups, a moderation effect seemed to be present. For studying the moderation, i.e., how the change in serial STM with age is related to the change in language competence, we ran a series of regression analyses with interaction terms.

### Predicting Expressive Language, Receptive Language, and Language Reasoning

A model with non-verbal reasoning as control variable, age, group (TD = 0, DLD = 1), age × group, serial STM, age × serial STM, group × serial STM, and age × group × serial STM as explanatory variables was used to account for variation in the three first-level factor composites: expressive and receptive language as well as language reasoning. Table 4 presents unstandardized (b_i_) and standardized (β_i_) regression coefficients for each effect.

**Table 4:** Results of the multiple regression analyses predicting language composites from centered age (in months), group status, non-verbal serial short-term memory, their interactions and non-verbal reasoning.

	**b**_**i**_	**β**_**i**_	***p***^**a**^
**Expressive language composite (*****R***^**2**^ **=** **0.72)**			
Non-verbal reasoning	0.14	0.14	0.136
Age	0.03	0.31	0.001
DLD	−1.26	−0.67	< 0.001
Age × DLD	0.02	0.13	0.130
Non–verbal serial STM	−0.06	−0.05	0.701
Age × Non-verbal serial STM	0.00	0.00	0.996
DLD × Non-verbal serial STM	−0.26	−0.12	0.450
Age × DLD × Non-verbal serial STM	0.01	0.06	0.679
**Receptive language composite (*****R***^**2**^ **=** **0.76)**			
Non-verbal reasoning	0.17	0.18	0.044
Age	0.04	0.51	< 0.001
DLD	−1.09	−0.62	< 0.001
Age × DLD	0.03	0.16	0.025
Non-verbal serial STM	−0.15	−0.14	0.121
Age × Non-verbal serial STM	0.02	0.22	0.025
DLD × Non-verbal serial STM	−0.31	−0.15	0.142
Age × DLD × Non-verbal serial STM	0.06	0.28	0.010
**Complex language reasoning composite (*****R***^**2**^ **=** **0.83)**			
Non-verbal reasoning	0.09	0.09	0.186
Age	0.04	0.43	< 0.001
DLD	−1.31	−0.71	< 0.001
Age × DLD	0.02	0.12	0.033
Non-verbal serial STM	−0.01	−0.01	0.907
Age × Non-verbal serial STM	0.01	0.06	0.463
DLD × Non-verbal serial STM	−0.11	−0.05	0.622
Age × DLD × Non-verbal serial STM	0.03	0.14	0.139

*DLD, Dummy variable, before centering; 0, Typically developing children; 1, Children with developmental language disorder and after centering −0.453 and 0.547, respectively; STM, Short-term memory*.

a *p-values were calculated using heteroskedasticity-consistent standard error estimators (HC4, Cribari-Neto and Zarkos, 2004; Hayes and Cai, 2007) and small-sample degrees of freedom for multiple imputations (Reiter, 2007; Van Ginkel and Kroonenberg, 2014)*.

The model for the expressive language composite was statistically significant (*F*_*8,106*_ = 35.1, *p* < 0.001), but in this model only the effects of age and group were significant (see Table 4).

**Figure 2:**
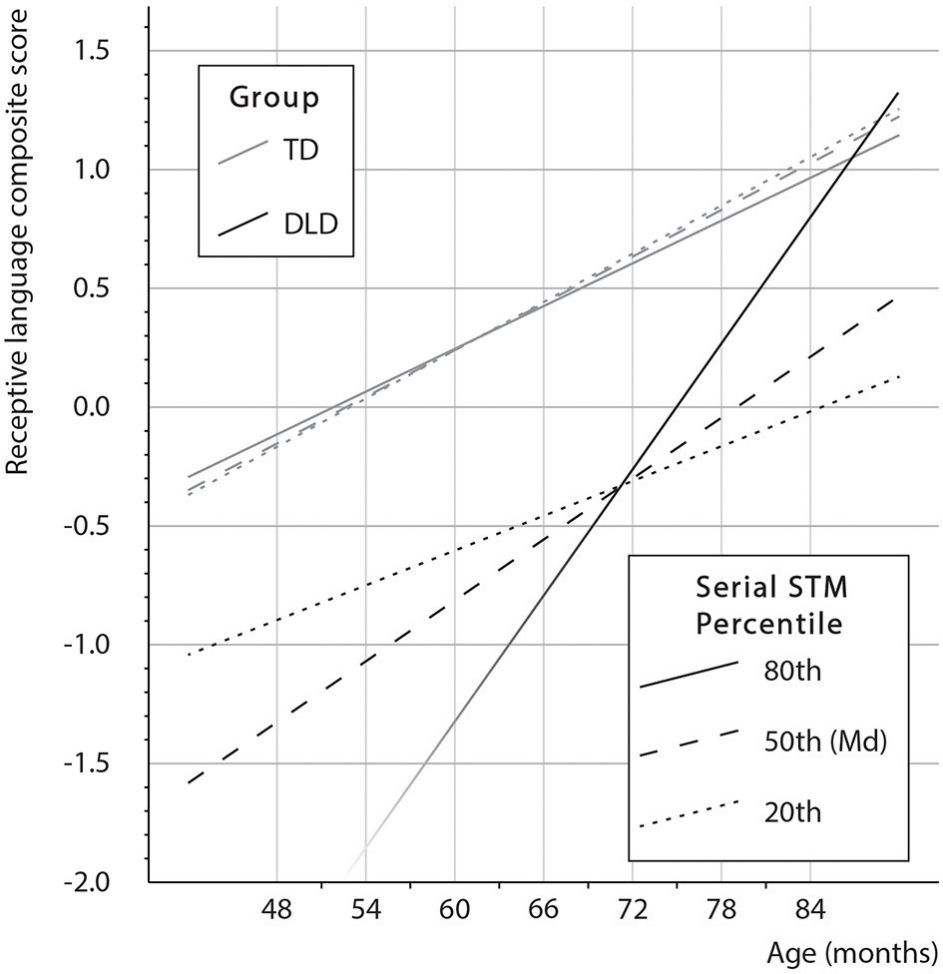
Visualization of the receptive language composite by age × group × serial STM interaction. The lower part of the line of the 80th percentile in the group with DLD is faded to indicate that this portion of the line for children under 60 months old is extrapolated. The regression model suggests that DLD children in the highest STM percentile are catching up with the TD children. Non-verbal reasoning was statistically controlled at the total sample mean = 0.

When predicting receptive language (*F*_*8,106*_ = 40.6, *p* < 0.001), the effects of non-verbal reasoning, age, and group, as well as the two-way interactions of age × group and age × serial STM and, importantly, the three-way interaction of interest: age × group × serial STM, were statistically significant (Table 4). The age × group × serial STM interaction suggests that, as a function of age, the relationship of non-verbal serial STM with receptive language differed between children with DLD and the TD children. This effect, along with other effects of the model, is presented in Figure 2. As the serial STM composite is a quantitative variable, we chose three percentile values (20th, 50th, and 80th) to demonstrate the interaction and be probed in the figure. In the figure, it can be seen that the effect of age on receptive language does not differ with serial STM performance in the TD children. In contrast, for the children with DLD, better non-verbal serial STM performance is associated with steeper growth of receptive language competence with age. Considering each group separately in a follow-up analysis of conditional effects, one can ask the critical question of whether there is a two-way interaction of age × serial STM in each group.

Different age × serial STM interactions in TD children and in children with DLD were suggested by the tests conditionally in each group (*b*_*cond*.*Age* × *serial STM*_ = *b*_*Age*× *serial STM*_ + *b*_*Age* × *group* × *serial STM*_ × *centered group value* = −0.003, *p* = 0.530 in the TD group and *b*_*cond*.*Age* × *serial STM*_ = 0.055, *p* = 0.012 in the group with DLD). In TD children, this suggests no two-way interaction, whereas in children with DLD there is an indication of an interaction. This is a test for the effect presented in Figure 2, showing that for children with DLD, good serial STM capacity seems to be associated with greater receptive language build-up between the ages of 4 and 6 years.

A regression model with the same predictor variables also significantly accounted for the variation in the language reasoning composite (*F*_*8,106*_ = 82.4, *p* < 0.001). For this outcome variable, the effect of non-verbal reasoning was not statistically significant, but the effects of age and group and their interaction were (Table 4). The three-way interaction age × group × serial STM showed a similar trend as the corresponding interaction for the receptive language but did not reach statistical significance (*p* = 0.139).

### Effects on Receptive Language When Controlling for the Two Other Language Composites

The three-way interaction of age × group × serial STM turned out to be important when accounting for the variation in the receptive language composite. Next, we tested whether the STM moderation effect held when also the two other language composites were considered in the model. These results are presented in Table 5. The model was statistically significant (*F*_*10,104*_ = 43.9 and *p* < 0.001) and the three-way interaction (age × group × serial STM) was also significant. In this model, the conditional age × serial STM effect in the TD group was again not statistically significant (*b*_*cond*.*Age* × *serial STM*_ = 0.001, *p* = 0.895), whereas it was in the group with DLD (*b*_*cond*.*Age* × *serial STM*_ = 0.041, *p* = 0.020). This again indicates that in the group with DLD, better serial STM was associated with steeper receptive language growth with age compared to the language development in those with poorer serial STM.

**Table 5:** Results of the multiple regression analyses predicting receptive language composite from centered age (in months), group status, serial short-term memory, their interactions, non-verbal reasoning, and two other language composites.

	**b**_**i**_	**β**_**i**_	***p***^**a**^
**Receptive language composite (*****R***^**2**^ **=** **0.83)**			
Non-verbal reasoning	0.11	0.12	0.107
Expressive language composite	0.04	0.05	0.692
Complex language reasoning composite	0.57	0.59	< 0.001
Age	0.02	0.24	0.003
DLD	−0.29	−0.16	0.104
Age × DLD	0.01	0.08	0.175
Non-verbal serial STM	−0.14	−0.13	0.085
Age × Non-verbal serial STM	0.02	0.18	0.027
DLD × Non–verbal serial STM	−0.24	−0.11	0.193
Age × DLD × Non-verbal serial STM	0.04	0.20	0.030

*DLD, Dummy variable, before centering; 0, Typically developing children; 1, Children with developmental language disorder; STM, Short–term memory*.

a *p-values were calculated using heteroskedasticity-consistent standard error estimators (HC4, Cribari-Neto and Zarkos, 2004; Hayes and Cai, 2007) and small-sample degrees of freedom for multiple imputations (Reiter, 2007; Van Ginkel and Kroonenberg, 2014)*.

### Effects of Age and DLD on Non-verbal Serial STM

**Figure 3:**
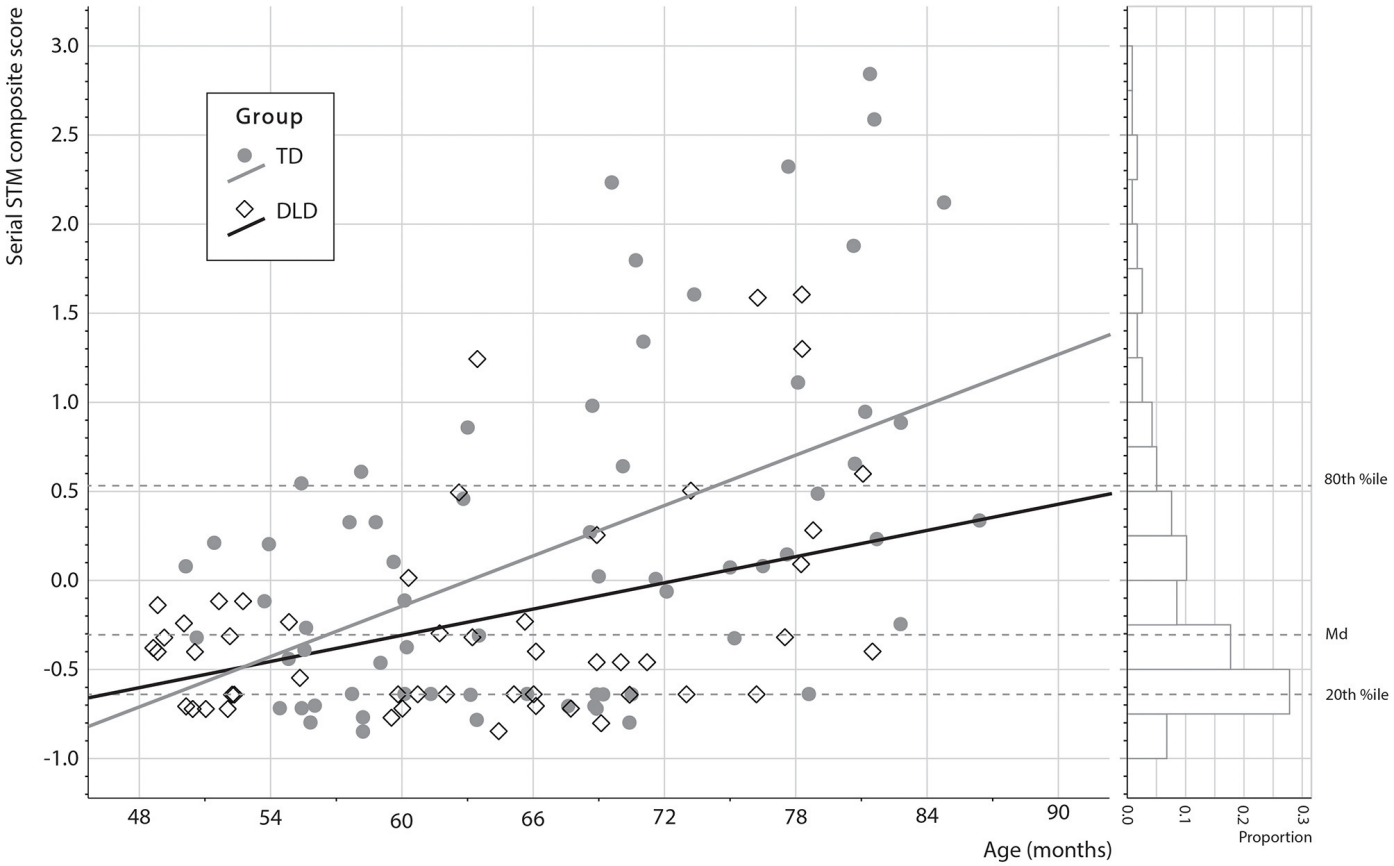
Visualization of the serial STM composite score by age × group. The 80th, 50th (Md = Median) and 20th percentile values are marked as dashed light gray horizontal lines. The regression model suggests steeper STM development in the TD group.

Non-verbal serial STM moderated the cross-sectional acquisition of receptive language in children with DLD. Therefore, we went on to investigate how age and group predicted non-verbal serial STM capacity. The results of this analysis are presented in Table 6. The model was statistically significant (*F*_4,110_ = 11.4 and *p* < 0.001). Importantly, the age × group interaction was significant (*p* = 0.042). The conditional effect of age on serial STM was statistically significant only in the TD group (*b*_*cond*.*Group*_ = 0.028, *p* = 0.010) and not in the group with DLD (*b*_*cond*.*Group*_ = 0.001, *p* = 0.911), suggesting significant serial STM improvement with age only in the TD group. In this model, the effect of non-verbal reasoning was particularly large. It is possible that controlling for this variable in this case weakened other effects unnecessarily (Dennis et al., 2009; Earle et al., 2017). When we tested the model without controlling for non-verbal reasoning, the conditional effect of age on non-verbal serial STM was statistically significant in both TD children (*b*_*cond*.*Group*_ = 0.047, *p* < 0.001) and in the group with DLD (*b*_*cond*.*Group*_ = 0.024, *p* = 0.006). These results are shown in Figure 3.

**Table 6:** Results of the multiple regression analysis predicting non-verbal serial short-term memory from centered age (in months), group status, their interaction and non-verbal reasoning.

	**b**_**i**_	**β**_**i**_	***p***^**a**^
**Non-verbal serial STM composite (*****R***^**2**^ **=** **0.34)**			
Non-verbal reasoning	0.35	0.39	0.001
Age	0.02	0.20	0.059
DLD	−0.10	−0.06	0.460
Age × DLD	−0.03	−0.17	0.042

*DLD, Dummy variable, before centering; 0, Typically developing children; 1, Children with developmental language disorder; STM, Short-term memory*.

a *p-values were calculated using heteroskedasticity-consistent standard error estimators (HC4, Cribari-Neto and Zarkos, 2004; Hayes and Cai, 2007) and small-sample degrees of freedom for multiple imputations (Reiter, 2007; Van Ginkel and Kroonenberg, 2014)*.

## Discussion

We hypothesized that serial STM performance is related to the cross-sectionally studied development of language competence, specifically expressive and receptive language and language reasoning, in 4–6-year-old Finnish DLD and TD children. We developed two non-verbal serial STM tasks to avoid measuring serial STM for order with a language-based task. We discovered that, with non-verbal reasoning ability controlled, serial STM moderated the relationship between age and receptive language in children with DLD: better non-verbal serial STM was associated with greater age-gains in the receptive language in children with DLD but not in the group of TD children. Comparable moderation effects were not detected for expressive language or language reasoning. The moderation effect in receptive language remained even after statistically controlling for the two other language composites. We further found that the development of non-verbal serial STM with age was slower in DLD than in TD children in our cross-sectional samples. Thus, it can be speculated that this serial order STM limitation may have impaired the acquisition of receptive language skills in children with DLD.

Our hypothesis of the relevance of serial order STM for language development stemmed from the research of vocabulary development and learning in TD children (Majerus et al., 2006a,b; Majerus and Boukebza, 2013; Attout et al., 2020). This research was conducted using verbal STM tasks tapping item and order memory separately. However, the possibility of a domain-general serial order mechanism playing a role in STM and developmental language disorders, such as dyslexia and DLD, has been suggested by Cowan et al. (2017), who studied 7–9-year-old children with only dyslexia or dyslexia combined with DLD. Further, Majerus (2019) has recently suggested that both domain-general and domain-specific serial order processing may be involved in the representation of verbal serial order information. In line with our hypotheses, the results of the present study suggest that order processing in the STM of children with DLD is, indeed, not as efficient as in TD children, and that this may be related to their slower language development.

However, such a picture was robustly seen only for our receptive language composite. The failure to find a similar effect for expressive language was surprising. An inspection of our data suggests that this null result was driven by a number of the older children with DLD doing relatively better than their younger peers in receptive language tasks, whereas such good performance was absent in the expressive tasks. The lack of high scores in the expressive tests used here may be an artifact of the individual tasks being variants of picture naming. Perhaps children with DLD were more reluctant to do these tasks because the tasks required them to respond verbally (cf., 0-scores and missing data for children in the DLD group in Table 1). This tendency could be influencing also the results of those children with DLD that did the tasks but, perhaps, gave up too quickly. This could be a sample-specific validity difficulty, and further studies are needed to determine whether the different patterns for receptive and expressive language can be replicated and whether these patterns vary depending on age. In a parallel sample from the HelSLI project, we tested children with an immigrant background who are developing a second language. We found the patterns of receptive compared to expressive language to be more similar to each other in that sample (Lahti-Nuuttila et al., submitted for publication). The present study included mostly lexical tasks. Future studies could focus on differentiating between language dimensions (e.g., vocabulary, syntax) and their associations with non-verbal serial STM.

One explanation for these results is that, in addition to pSTM, also non-phonological serial order STM impacts language acquisition. For at least some children with DLD, the development of general serial order STM appears to be delayed. Together with more specific problems in pSTM (i.e., verbal serial order STM), this could result in substantive constraints on their language acquisition. Alternatively, verbal and non-verbal serial STM might both depend on similarly implemented domain-bound (Endress, 2019) serial ordering mechanisms in addition to possible domain-specific mechanisms, such as internal speech or visual imagery. For future research, we want to put forward the hypothesis that performance in verbal STM tasks relies on three components: a domain-bound copy of a general mechanism for representing temporal order, ability to represent the domain-specific content (e.g., syllables) that have to be ordered, and structured language knowledge already present in LTM providing top-down support (cf., Isbilen et al., 2020). So far, the efficiency of pSTM has been thought to depend mainly on phonological content representation. However, an impairment of a domain-general temporal structuring mechanism could affect the ability to represent the order of phonemes and syllables in both STM and word learning (cf., Gupta and Tisdale, 2009), and result in slower accumulation of verbal chunks in LTM. As language development proceeds, top-down linguistic knowledge comes to play an increasing role in both verbal STM and new learning of linguistic material, decreasing reliance on the ordering mechanism and changing the causal drivers of language acquisition (cf., Gathercole et al., 1992).

According to Majerus et al. (2006a,b), Leclercq and Majerus (2010), Martinez Perez et al. (2012), and Majerus and Boukebza (2013), children with compromised serial STM processing capacities may not be capable of mentally rehearsing the phonemic pattern of a new word as easily as children with better serial STM processing. One possible underlying reason could be the efficiency of a domain-general serial ordering mechanism. Current theoretical models of serial order processing suggest that similar principles underlie order processing in different domains (Hurlstone et al., 2014). Recent empirical data suggest that serial order in different domains may be supported by a time-dependent linear context signal (Hurlstone and Hitch, 2015, 2018; Hurlstone, 2019). However, a special feature of the verbal domain is that it additionally appears to allow nested order signals within temporal groups (Hurlstone and Hitch, 2015, 2018; Hurlstone, 2019). Our tasks, as well as the verbal STM tasks that have been associated with vocabulary development in previous research (Majerus et al., 2006a,b; Leclercq and Majerus, 2010; Martinez Perez et al., 2012; Majerus and Boukebza, 2013), do not involve explicit temporal grouping structure. Thus, rather than revealing domain-specific recursive temporal ordering effects, these tasks may predominantly rely on a list-level linear context signal for order representation, for instance, constantly decreasing signal strength from the beginning of the list toward the end of the list. Targeted experiments are needed to explore further whether more than one ordering mechanism is at play with language material.

Although there was clear evidence that non-verbal serial STM capacity increased with age in TD children, this increase did not seem to have a moderating effect on their language competence. It could be that the moderation effect is related to a particular stage of language acquisition and might be found in younger TD children. Our receptive language tests may also be less sensitive to improvement among TD children. Whether the interaction discovered here could result from TD children having advanced more in their STM development, in their language development, in both, or in the development of some other domain (e.g., attention) needs to be further studied.

Also, intact domain-specific phonological processes could have large and more specific effects during typical development, reducing the order STM moderation effect in TD children. Whether phonological development is independent of order STM requires further study. Studying non-verbal serial STM in children with other developmental disorders would be valuable. For example, Cowan et al. (2017), in their study, suggested that children with only dyslexia and children with both dyslexia and DLD did not differ in serial order STM, but the dyslexia+DLD group was more challenged by a non-word repetition task measuring phonological memory. It is possible that the difference between the two groups reflects severity rather than qualitatively different deficits. These researchers did not have a group with DLD only (i.e., without dyslexia), so research that would also regard this distinction would be needed.

The non-verbal serial STM tasks for this study were devised with practical considerations in mind. Because the children with DLD were studied as part of their already busy examination visits at the hospital, the tasks could not take long. The small number of trials probably limited test reliability. Twenty—especially younger—children in the TD group and 20 in the DLD group did not succeed at the tasks even with the shortest series of two stimuli. Thus, guessing or momentary attentional lapses in a “same-different” task could have increased error variance. Lower reliability of the tasks can be expected to have attenuated rather than increased the reported effects. It is essential to improve the tasks in the future to be more reliable even with younger children. This could be achieved with optimal task parameters (e.g., presentation rate) and presentation of more trials.

Our complex stimuli also presented an attentional load that could have resulted in poorer STM capacity estimates (Astle et al., 2012; Archibald et al., 2015; Rhodes and Cowan, 2018). However, in line with dynamic attention theory (Jones, 1976, 2019), we suspect that time-based attention may be an inherent component of serial STM tasks rather than a competing explanation. In the future, improving the reliability of non-verbal serial STM measures and comparing them to the tasks that have been used in studying verbal order STM is worth pursuing.

We studied the non-verbal serial STM as a domain-general process, common to visual and auditory modality. This interest was based on previous research (Laasonen et al., 2012; Cowan et al., 2017; Majerus, 2019) but also on comparable associations of the visual and auditory tasks to other variables in this study. Statistical learning (SL) is another cognitive ability thought to be related to language development (Frost et al., 2015; Bogaerts et al., 2021). Research on SL (Conway and Christiansen, 2005, 2006) has shown how modality-specific mechanisms interact with a possibly general, but domain-bound (Endress, 2019), mechanism to produce both differences and similarities in performance. This is similarly a research prospect that is important to explore. The results from SL studies also highlight the necessity to consider using different temporal parameter values for different presentation modalities (Arciuli and Simpson, 2011; Emberson et al., 2011).

The different inclusion criteria for PIQ may raise concerns about the generalizability of our results. Finding TD children with PIQ below 85 without comorbidities would have been hard. In our DLD sample, assessed with complete IQ tests, they were common. However, the overlap of the DLD and TD groups in PIQ distributions extends over two standard deviations. Using the non-verbal reasoning composite as a covariate in the analyses increased this overlap further, alleviating this concern.

## Summary and Conclusions

To summarize, we developed two novel tasks to test serial STM in DLD without using verbal material. The tasks were administered to a group of 4–6-year-old children with DLD and their TD controls. Our results indicated that serial STM improves more slowly with age in children with DLD than in TD children. Furthermore, better serial STM was related to larger age gains in the receptive language in the DLD, but not the TD, group. These results highlight the relevance of non-verbal serial STM as a domain-general factor but are also compatible with the prevailing models of DLD that see this disorder as causally complex, with both domain-general and domain-specific origins (Archibald and Joanisse, 2013; Archibald and Harder Griebeling, 2016). Our findings are among the first investigating non-verbal serial STM and DLD. Although more research is required, our results suggest that the assessment of serial non-verbal STM could advance the identification of DLD, and especially so when the verbal assessment of the child is for some reason not valid or reliable enough. An example of such a situation is assessing a bilingual child when assessment in the child’s first language is not possible.

## Data Availability Statement

The datasets generated for this article are not readily available because of privacy and ethical restrictions. Requests to access the datasets should be directed to marja.laasonen@helsinki.fi.

## Ethics Statement

The studies involving human participants were reviewed and approved by the Ethical board of Helsinki University Hospital. Written informed consent to participate in this study was provided by the participants’ legal guardian/next of kin.

## Author Contributions

EA, ES, ML, SK, and SS contributed to conception and design of the HelSLI project. ES and ML contributed to conception and design of this study. ML, PL-N, and SS organized the database. ES, ML, and PL-N performed the statistical analysis and wrote the first draft of the manuscript. EA and SS wrote sections of the manuscript. All authors contributed to manuscript revision, read, and approved the submitted version.

## Conflict of Interest

The authors declare that the research was conducted in the absence of any commercial or financial relationships that could be construed as a potential conflict of interest.

## Footnotes

^1^MLMV estimator uses only non-missing observations so the data from the children with missing values could not be used. To improve the CFA, we also used observations from children that had scores for the language variables but could not be included in the study because they did not take part in the nonverbal serial STM tasks. Thus, the sample size for the factor analysis was 143.

## Supplementary Material

Supplementary Figure 1 **(A,B)** Modified bean plot of distributions of age and composite variables. Dots represent individual children and areas represent the probability density with an Epanechnikov kernel function. Means, standard deviations, and quartiles are also marked. N.B. Different scaling on variables.

Supplementary Figure 2 Total sample CFA model of the language variables. BNT, Boston Naming Test; RDLS, Reynell Developmental Language Scales III; EOWPVT, Expressive One Word Picture Vocabulary Test; WPPSI, Wechsler Preschool and Primary Scale of Intelligence, Third edition; Pict. Naming, Picture Naming; Receptive Voc., Receptive Vocabulary; ROWPVT, Receptive One Word Picture Vocabulary Test; Word Reas., Word Reasoning; Nepsy-II Compr. Instr., Comprehension of Instructions subtest from the Nepsy-II.

Supplementary Table 1 Correlation matrix of variables.

Supplementary Movie Clip 1 Non-verbal serial STM task demonstration. Time 0:00–2:21 visual task starts with four trials with sequences of two stimuli and these are followed by four trials with sequences of three stimuli, time 2:22–3:16 auditory task with four trials with sequences of two stimuli.

## Appendix 1

**Table:** List of Language and cognitive tests and their role in the study.

***Wechsler Primary and preschool test of intelligence III (WPPSI-III)***		
Information	Child responds to a question by choosing a picture from four response options and answers questions that address a broad range of general knowledge topics.	Complex language reasoning
Vocabulary	Child names pictures and gives definitions for words.	Complex language reasoning
Word reasoning	Child identifies the common concept that is described in a series of increasingly specific clues.	Complex language reasoning
Block design	While viewing a constructed model or a picture of a one the child assembles alike design with one- and two-color cubic blocks within a time limit.	Non-verbal reasoning
Matrix reasoning	Child looks at an incomplete matrix of images and selects for the missing part the completing image from 4 or 5 alternatives.	Non-verbal reasoning
Receptive vocabulary	Child looks at a group of four pictures and points to the one the tester names aloud.	Receptive language
Picture naming	Child names pictures that are displayed to her/him.	Expressive language
***Nepsy II***		
Comprehension of instructions	Child follows verbal instructions that at start have quite simple structure but become more complex.	Complex language reasoning
Sentence repetition	Child repeats descriptive sentences starting from short and simple sentences but becoming longer and more complex.	Non-verbal Serial STM validation
***Leiter-R***		
Forward memory	Tester points a sequence of images of objects and after that the child points them in the same order.	Non-verbal Serial STM validation
***Reynell Developmental Language Scales III—RLDS III***		
comprehension scale	Child manipulates a toy or points to a picture or a toy according different tasks that the tester gives to the child.	Receptive language
Expressive scale	Child names toys or pictures or tells about the events the tester performs with toys or the events that are illustrated.	Expressive language
***Boston Naming Test***		Expressive language
	Child names pictures that are displayed to her/him.	
***Expressive One-Word Picture Vocabulary Test 4 (EOWPVT-4)***		Expressive language
	Child names pictures that are displayed to her/him.	
***Receptive One-Word Picture Vocabulary Test 4 (ROWPVT-4)***		Receptive language
	Child looks at a group of four pictures and points to the one the tester names aloud.	
