# Supplementary material for: Short-Term Memory for Serial Order Moderates Aspects of Language Acquisition in Children With Developmental Language Disorder: Findings From the HelSLI Study
Source: Front Psychol. 2021 Apr 20;12:608069. doi: 10.3389/fpsyg.2021.608069 (PMC8096175; doi:10.3389/fpsyg.2021.608069)
Supplement: Supplementary Figure 1 — (A,B) Modified bean plot of distributions of age and composite variables. Dots represent individual children and areas represent the probability density with an Epanechnikov kernel function. Means, standard deviations, and quartiles are also marked. N.B. Different scaling on variables. [file Presentation_1.pdf]

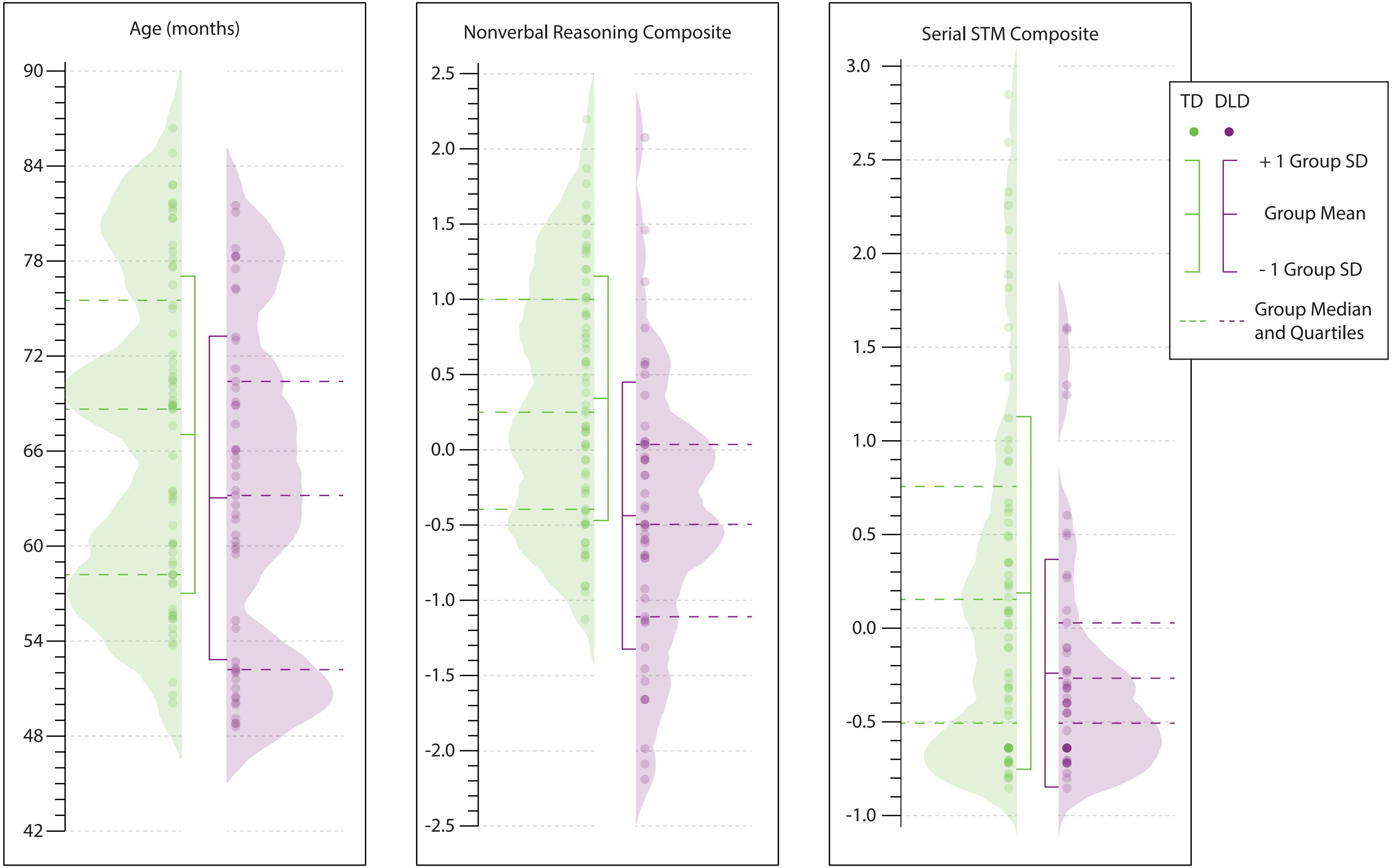

Supplementary Figure 1a. Modified bean plot of distributions of age and nonverbal reasoning and serial short-term memory composite variables. Dots present individual children and areas represent the probability density with epanechnikov kernel function. Means, standard deviations and quartiles are also marked. N.B. Different scaling on variables.

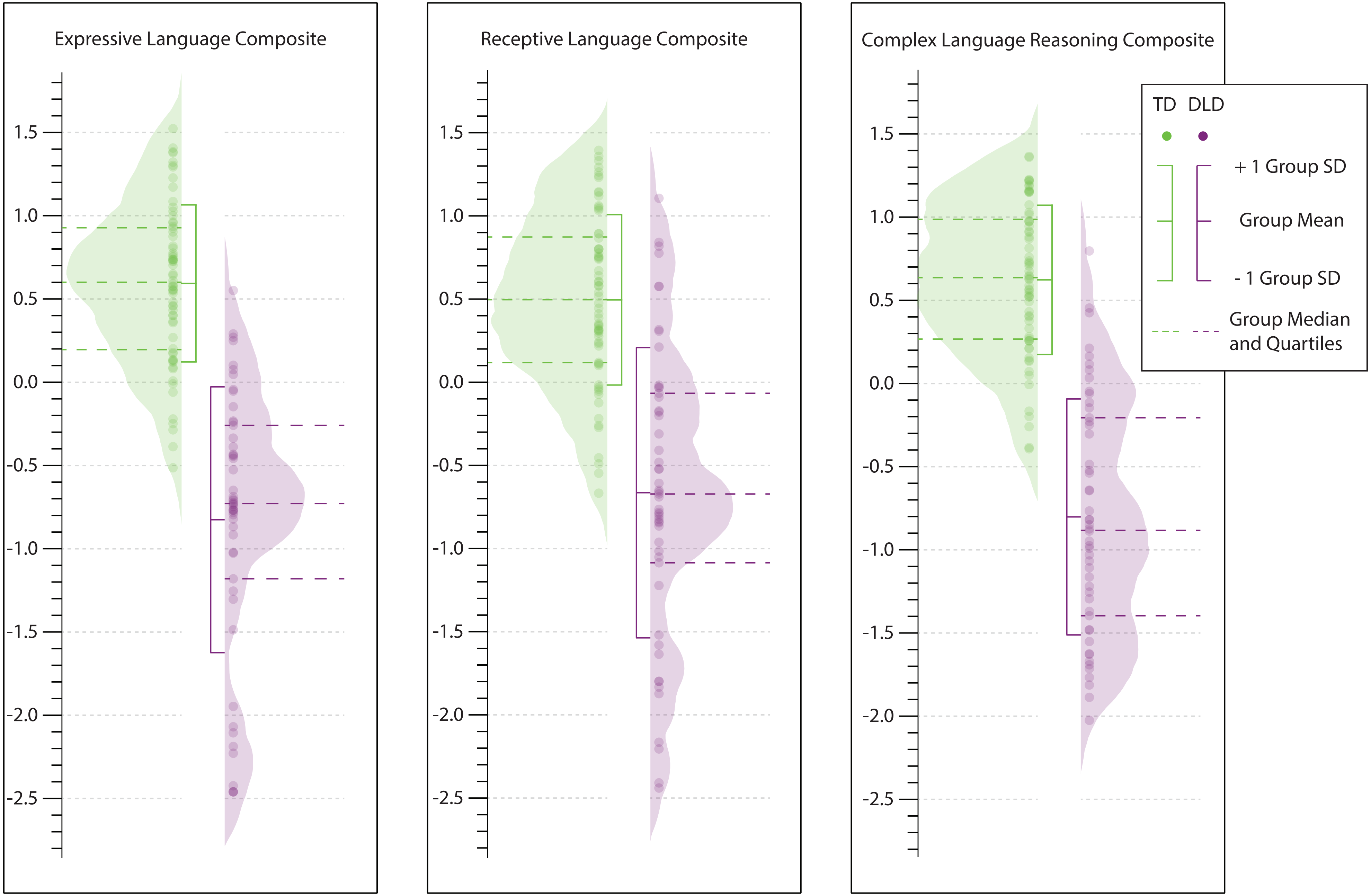

Supplementary Figure 1b. Modified bean plot of distributions of three language composite variables. Dots present individual children and areas represent the probability density with epanechnikov kernel function. Means, standard deviations and quartiles are also marked.

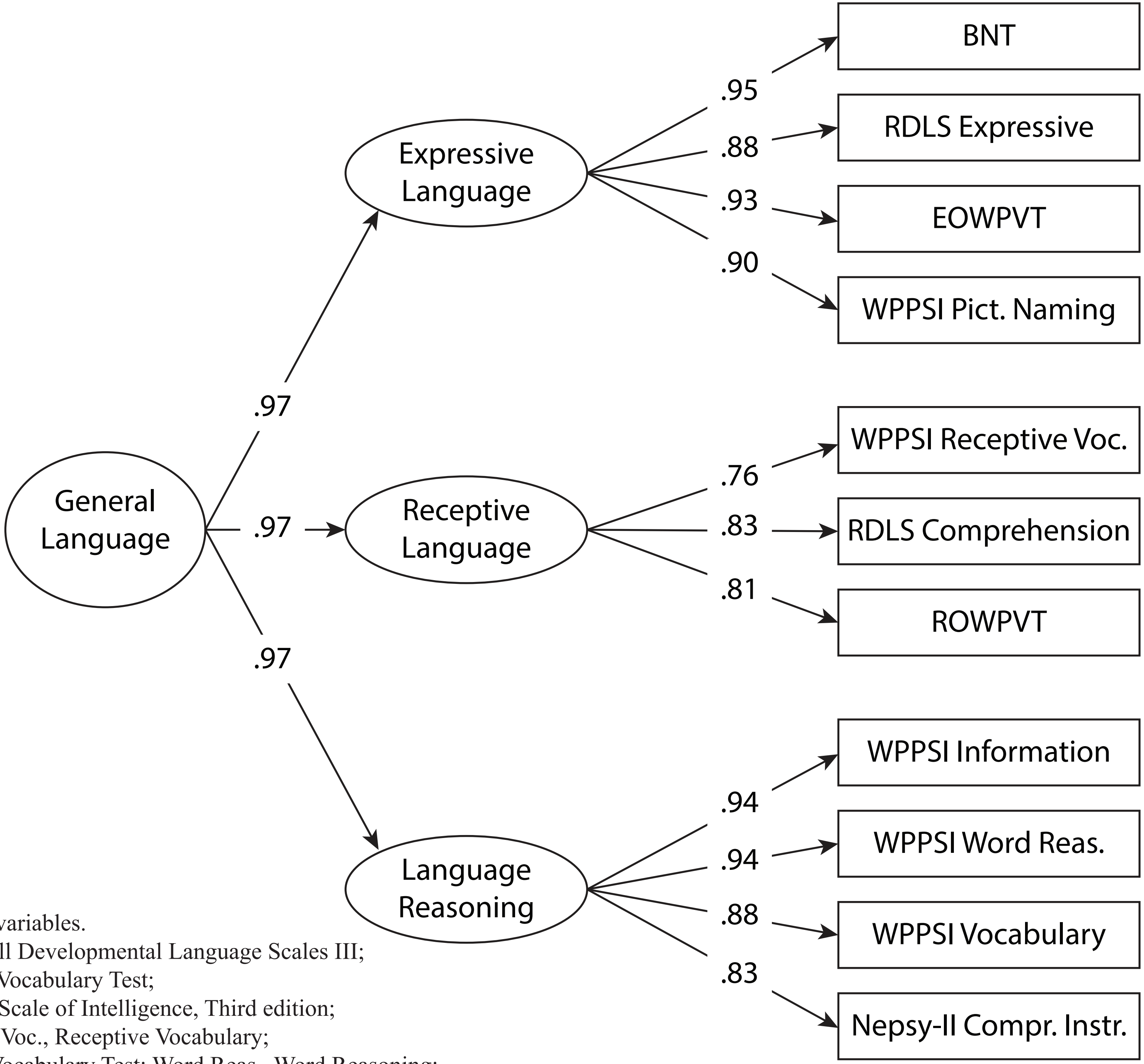

Supplementary Figure 2.
Total sample CFA model of the language variables.
BNT, Boston Naming Test; RDLS, Reynell Developmental Language Scales III;
EOWPVT, Expressive One Word Picture Vocabulary Test;
WPPSI, Wechsler Preschool and Primary Scale of Intelligence, Third edition;
Pict. Naming, Picture Naming; Receptive Voc., Receptive Vocabulary;
ROWPVT, Receptive One Word Picture Vocabulary Test; Word Reas., Word Reasoning;
Nepsy-II Compr. Instr., Comprehension of Instructions subtest from the Nepsy-II.
