# Supplementary material for: Short-Term Memory for Serial Order Moderates Aspects of Language Acquisition in Children With Developmental Language Disorder: Findings From the HelSLI Study
Source: Front Psychol. 2021 Apr 20;12:608069. doi: 10.3389/fpsyg.2021.608069 (PMC8096175; doi:10.3389/fpsyg.2021.608069)
Supplement: Supplementary Table 1 — Correlation matrix of variables. [file Table_1.pdf]

Supplementary Table 1. *Correlation matrix of variables. Total group correlations are below, and group-wise correlations are above the diagonal. Pooled from 20 multiple imputation datasets.*

													TD<br>DLD																		
	1.	2.	3.	4.	5.	6.	7.	8.	9.	10.	11.	12.	13.	14.	15.	16.	17.	18.	19.	20.	21.	22.	23.	24.	25.	26.	27.	28.	29.	30.	31.
1. Age (months)		.67***<br>.76***	.52***<br>.72***	-.11<br>.08	.66***<br>.72***	-.03<br>-.03	.52***<br>.67***	.01<br>.12	.64***<br>.63***	.11<br>.21	.58***<br>.71***	-.01<br>.32*	.51***<br>.68***	-.12<br>.26	.54***<br>.61***	.37**<br>.55***	.62***<br>.60***	.34**<br>.42**	-.10<br>-.07	.55***<br>.42**	-.17<br>-.01	.54***<br>.69***	.70***<br>.70***	.50***<br>.58***	.51***<br>.66***	.46***<br>.36**	.37**<br>.33*	.50***<br>.41*	.56***<br>.59***	.76***<br>.68***	.72***<br>.75***
2. Nonverbal Reasoning[a,b]	.71***		.88***<br>.94***	.54***<br>.62***	.90***<br>.94***	.58***<br>.52***	.28*<br>.64***	-.08<br>.26	.45***<br>.56***	.13<br>.33*	.30*<br>.64***	-.09<br>.40**	.41***<br>.71***	.00<br>.44**	.45***<br>.51***	.37**<br>.41**	.55***<br>.58***	.34**<br>.39**	.07<br>.01	.40***<br>.47***	-.08<br>.20	.46***<br>.60***	.54***<br>.66***	.50***<br>.58***	.56***<br>.65***	.53***<br>.34*	.39**<br>.38**	.56***<br>.43**	.50***<br>.52***	.59***<br>.65***	.46***<br>.71***
3. Matrix Reas., Raw Score	.63***	.93***		.77***<br>.72***	.59***<br>.77***	.31*<br>.34*	.19<br>.59***	-.11<br>.22	.36**<br>.48***	.10<br>.31*	.21<br>.57***	-.11<br>.36**	.32**<br>.64***	-.03<br>.39**	.41**<br>.46***	.39**<br>.37**	.45***<br>.52***	.38**<br>.33*	.17<br>.01	.39**<br>.45***	.02<br>.18	.43***<br>.49***	.40***<br>.59***	.45***<br>.46***	.41***<br>.54***	.43***<br>.28*	.29*<br>.33*	.44***<br>.36*	.48***<br>.46***	.50***<br>.57***	.34**<br>.63***
4. Matrix Reas., Std. Score[a]	.05	.64***	.79***		.21<br>.44**	.41**<br>.56***	-.17<br>.26	-.13<br>.25	-.05<br>.10	.06<br>.29*	-.21<br>.19	-.14<br>.25	.01<br>.32*	.07<br>.40**	.07<br>.08	.19<br>-.01	.08<br>.17	.22<br>.07	.31*<br>.11	.03<br>.29*	.15<br>.35*	.10<br>.07	-.03<br>.23	.14<br>.12	.10<br>.15	.20<br>.14	.09<br>.15	.18<br>.17	.15<br>.09	.03<br>.22	-.14<br>.24
5. Block Design, Raw Score[a]	.70***	.93***	.72***	.40***		.71***<br>.65***	.30*<br>.62***	-.05<br>.26	.44***<br>.59***	.13<br>.30*	.32**<br>.62***	-.06<br>.40**	.41***<br>.70***	.02<br>.43**	.39***<br>.51***	.28*<br>.40**	.52***<br>.59***	.23<br>.40**	-.04<br>.01	.33**<br>.44**	-.15<br>.19	.40***<br>.64***	.54***<br>.66***	.44***<br>.63***	.58***<br>.68***	.52***<br>.36**	.40***<br>.39**	.55***<br>.44***	.43**<br>.52***	.55***<br>.65***	.47***<br>.71***
6. Block Design, Std. Score[a]	.03	.61***	.41***	.54***	.72***		-.09<br>.21	-.09<br>.28*	-.01<br>.18	.09<br>.27	-.14<br>.18	-.09<br>.24	.07<br>.29*	.13<br>.37**	.01<br>.14	.02<br>.05	.11<br>.24	.01<br>.17	.07<br>.16	-.07<br>.20	-.03<br>.32*	.03<br>.19	.09<br>.20	.09<br>.32*	.32**<br>.23	.28*<br>.12	.20<br>.17	.29*<br>.17	.05<br>.16	.04<br>.23	-.05<br>.24
7. Vocab., Raw Score[a]	.51***	.54***	.50***	.26**	.51***	.23*		.85***<br>.79***	.45***<br>.74***	.20<br>.47**	.53***<br>.81***	.26*<br>.52***	.32**<br>.72***	-.01<br>.44***	.45***<br>.71***	.34**<br>.54***	.52***<br>.66***	.33**<br>.58***	.12<br>.10	.44***<br>.48***	.16<br>.22	.46***<br>.63***	.39**<br>.62***	.38**<br>.60***	.37**<br>.51***	.29*<br>.23	.16<br>.08	.28*<br>.19	.48***<br>.68***	.51***<br>.66***	.81***<br>.88***
8. Vocab., Std. Score[a]	.17	.33***	.32**	.29**	.29**	.25**	.92***		.15<br>.54***	.19<br>.49***	.26*<br>.50***	.30*<br>.42**	.07<br>.40**	.07<br>.35**	.18<br>.51***	.15<br>.39**	.23<br>.47***	.14<br>.52***	.16<br>.26	.15<br>.32*	.25*<br>.27	.19<br>.32*	.03<br>.24	.13<br>.35*	.17<br>.17	.06<br>-.01	-.01<br>-.16	.03<br>-.09	.21<br>.52***	.13<br>.35**	.52***<br>.59***
9. Inform., Raw Score[a]	.55***	.62***	.57***	.31**	.58***	.29**	.76***	.67***		.81***<br>.70***	.66***<br>.71***	.32**<br>.52***	.43***<br>.69***	.02<br>.44**	.76***<br>.75***	.56***<br>.70***	.73***<br>.76***	.53***<br>.76***	.22<br>.30*	.55***<br>.58***	.12<br>.27	.42***<br>.73***	.64***<br>.59***	.64***<br>.74***	.49***<br>.54***	.31**<br>.32*	.23<br>.12	.33**<br>.26	.76***<br>.82***	.68***<br>.74***	.77***<br>.88***
10. Inform., Std. Score[a]	.24**	.46***	.45***	.41***	.40***	.35***	.71***	.73***	.87***		.40***<br>.52***	.42***<br>.64***	.20<br>.49***	.15<br>.49***	.58***<br>.40**	.48***<br>.37**	.48***<br>.45***	.48***<br>.49***	.43***<br>.32*	.31*<br>.36**	.30*<br>.28*	.16<br>.41**	.35**<br>.30*	.47***<br>.47***	.28*<br>.33*	.12<br>.32*	.06<br>.09	.11<br>.25	.59***<br>.47***	.36**<br>.42**	.47***<br>.62***
11. Word Reas., Raw Score[a]	.53***	.61***	.57***	.33***	.55***	.28*	.80***	.72***	.86***	.80***		.78***<br>.76***	.40***<br>.76***	.05<br>.52***	.54***<br>.71***	.34**<br>.45**	.58***<br>.59***	.40**<br>.46**	.12<br>.08	.41***<br>.52***	-.01<br>.29*	.35**<br>.73***	.61***<br>.68***	.53***<br>.57***	.27*<br>.55***	.24<br>.35*	-.03<br>.09	.13<br>.26	.55***<br>.61***	.60***<br>.74***	.78***<br>.91***
12. Word Reas., Std. Score[a]	.24**	.42***	.42***	.36***	.37***	.30**	.73***	.75***	.77***	.86***	.90***		.15<br>.54***	.20<br>.51***	.23<br>.38**	.17<br>.11	.23<br>.31*	.29*<br>.29*	.29*<br>.11	.10<br>.47***	.12<br>.38**	.02<br>.50***	.27*<br>.49***	.27*<br>.25	-.04<br>.39**	.01<br>.35*	-.30*<br>.01	-.17<br>.22	.26*<br>.30*	.19<br>.56***	.43***<br>.66***
13. Compr. Instr., Raw Score	.57***	.67***	.61***	.37***	.62***	.33***	.68***	.56***	.77***	.67***	.80***	.68***		.78***<br>.81***	.38**<br>.62***	.46***<br>.53***	.47***<br>.58***	.28*<br>.44***	.07<br>.10	.32**<br>.58***	-.03<br>.41**	.29*<br>.72***	.53***<br>.67***	.52***<br>.66***	.44***<br>.65***	.23<br>.55***	.39**<br>.34*	.37**<br>.53***	.47***<br>.60***	.51***<br>.75***	.71***<br>.90***
14. Compr. Instr., Std. Score	.16	.41***	.39***	.40***	.37***	.37***	.52***	.55***	.59***	.65***	.65***	.67***	.87***		.06<br>.37**	.26*<br>.28	.11<br>.32*	.09<br>.24	.17<br>.14	-.01<br>.44**	.09<br>.51***	-.05<br>.45***	.12<br>.47***	.21<br>.39**	.14<br>.48**	-.04<br>.53***	.20<br>.27	.10<br>.48**	.15<br>.33*	.05<br>.52***	.29*<br>.64***
15. BNT, Raw Score	.53***	.60***	.58***	.34***	.54***	.28**	.76***	.67***	.88***	.79***	.84***	.73***	.73***	.57***		.58***<br>.73***	.80***<br>.85***	.67***<br>.77***	.43***<br>.43**	.61***<br>.40**	.26*<br>.14	.59***<br>.57***	.53***<br>.54***	.59***<br>.71***	.36**<br>.41**	.20<br>.24	.36**<br>.05	.34**<br>.17	.90***<br>.92***	.69***<br>.57***	.66***<br>.78***
16. RDLS Expr., Raw Score	.46***	.54***	.54***	.33***	.47***	.24*	.68***	.62***	.82***	.74***	.72***	.63***	.72***	.58***	.83***		.65***<br>.70***	.51***<br>.73***	.34**<br>.30*	.56***<br>.26	.35**<br>-.03	.42***<br>.50***	.48***<br>.33*	.59***<br>.76***	.29*<br>.35*	.32**<br>.21	.23<br>.11	.33**<br>.19	.81***<br>.87***	.60***<br>.43**	.54***<br>.63***
17. EOWPVT, Raw Score	.56***	.66***	.61***	.36***	.61***	.34***	.76***	.66***	.87***	.76***	.80***	.70***	.74***	.56***	.92***	.83***		.68***<br>.78***	.41***<br>.33**	.60***<br>.33*	.21<br>.07	.58***<br>.51***	.61***<br>.54***	.57***<br>.70***	.44***<br>.52***	.27*<br>.20	.25*<br>.11	.31*<br>.19	.92***<br>.92***	.74***<br>.52***	.73***<br>.72***
18. Pict. Naming, Raw Score[a]	.39***	.52***	.51***	.36***	.46***	.29**	.69***	.66***	.86***	.77***	.76***	.71***	.66***	.55***	.86***	.82***	.87***		.88***<br>.67***	.41***<br>.28*	.23<br>.06	.46***<br>.45***	.48***<br>.40**	.44***<br>.63***	.18<br>.29*	.20<br>.05	.17<br>.00	.23<br>.03	.80***<br>.91***	.56***<br>.43**	.48***<br>.62***
19. Pict. Naming, Std. Score[a]	.09	.35***	.39***	.44***	.26**	.31***	.60***	.66***	.67***	.78***	.64***	.72***	.52***	.56***	.75***	.67***	.70***	.86***		.15<br>.14	.31*<br>.13	.24<br>.14	.19<br>.05	.20<br>.20	-.03<br>-.14	.00<br>-.14	.02<br>-.18	.01<br>-.19	.56***<br>.48***	.23<br>.14	.16<br>.17
20. Receptive Voc., Raw Score[a]	.46***	.55***	.54***	.36***	.48***	.25*	.62***	.53***	.74***	.61***	.70***	.62***	.67***	.52***	.67***	.59***	.63***	.58***	.50***		.69***<br>.83***	.55***<br>.63***	.55***<br>.55***	.45***<br>.39**	.21<br>.33*	.25*<br>.29*	.20<br>.17	.27*<br>.27	.65***<br>.35*	.82***<br>.87***	.55***<br>.61***
21. Receptive Voc., Std. Score[a]	.04	.31***	.33***	.42***	.24*	.31***	.51***	.56***	.55***	.61***	.56***	.61***	.53***	.58***	.52***	.45***	.47***	.48***	.55***	.86***		.20<br>.35*	.07<br>.32*	.10<br>.16	-.16<br>.16	-.08<br>.21	-.05<br>.09	-.08<br>.18	.31*<br>.07	.33*<br>.61***	.09<br>.34*
22. RDLS Compr., Raw Score	.59***	.62***	.56***	.28**	.59***	.27**	.65***	.53***	.78***	.59***	.77***	.61***	.72***	.52***	.72***	.66***	.69***	.66***	.51***	.73***	.53***		.36**<br>.65***	.45***<br>.54***	.32**<br>.61***	.22<br>.31*	.35**<br>.05	.35**<br>.21	.60***<br>.56***	.71***<br>.90***	.50***<br>.79***
23. ROWPVT, Raw Score	.66***	.67***	.59***	.29**	.64***	.29**	.68***	.50***	.74***	.65***	.75***	.66***	.73***	.53***	.72***	.64***	.73***	.64***	.53***	.65***	.47***	.63***		.57***<br>.39**	.44**<br>.56**	.28*<br>.32*	.25*<br>.24	.32**<br>.33*	.62***<br>.49***	.87***<br>.80***	.67***<br>.72***
24. Sentence Repet., Raw Score	.47***	.62***	.58***	.39***	.57***	.37***	.75***	.68***	.87***	.82***	.83***	.76***	.78***	.65***	.85***	.85***	.73***	.82***	.69***	.65***	.54***	.70***	.69***		.39**<br>.53***	.28*<br>.35*	.34**<br>.22	.37**<br>.34*	.64***<br>.77***	.62***<br>.52***	.64***<br>.72***
25. Forward Mem., Raw Score	.56***	.69***	.60***	.34**	.67***	.40***	.66***	.56***	.73***	.66***	.71***	.61***	.74***	.59***	.66***	.61***	.74***	.59***	.44***	.54***	.40***	.68***	.67***	.73***		.33**<br>.38**	.47***<br>.37**	.49***<br>.45**	.38**<br>.43**	.43***<br>.57***	.52***<br>.64***
26. Visual serial STM	.44***	.47***	.39***	.23*	.48***	.26**	.30***	.15	.30**	.23*	.28**	.20*	.35***	.22**	.25**	.29**	.27**	.18	.10	.27**	.13	.27**	.32***	.29***	.35***		.37**<br>.42**	.83***<br>.85***	.29*<br>.19	.32**<br>.35*	.35**<br>.43**
27. Auditory serial STM	.38***	.44***	.37***	.20*	.44***	.26**	.29**	.18	.30**	.26**	.22*	.13	.42***	.33***	.34***	.30**	.31**	.23**	.18	.27**	.16	.27**	.35***	.36***	.47***	.40***		.82***<br>.84***	.30*<br>.07	.32**<br>.16	.27*<br>.19
28. Serial STM	.49***	.54***	.46***	.26**	.55***	.31***	.35***	.20*	.36***	.29**	.30**	.20*	.46***	.33***	.35***	.35***	.35***	.25**	.16	.32***	.17	.32***	.40***	.39***	.49***	.84***	.84***		.36**<br>.16	.39**<br>.30*	.37**<br>.37**
29. Expressive language	.51***	.62***	.59***	.37***	.55***	.30***	.77***	.69***	.91***	.81***	.83***	.73***	.75***	.60***	.96***	.92***	.96***	.94***	.79***	.65***	.51***	.72***	.72***	.89***	.68***	.26**	.31**	.34***		.76***<br>.54***	.71***<br>.75***
30. Receptive language	.64***	.69***	.64***	.35***	.65***	.30***	.73***	.58***	.85***	.70***	.84***	.71***	.80***	.59***	.79***	.71***	.78***	.71***	.58***	.90***	.70***	.89***	.86***	.77***	.71***	.32***	.34***	.40***	.79***		.73***<br>.82***
31. Language reasoning	.59***	.67***	.62***	.35***	.62***	.31***	.89***	.79***	.93***	.84***	.95***	.84***	.89***	.72***	.88***	.80***	.87***	.81***	.67***	.74***	.59***	.80***	.79***	.89***	.78***	.34***	.34***	.40***	.89***	.88***	

Note. TD = Typically developing children; DLD = Children with developmental language disorder; Matrix Reas. = Matrix Reasoning; Vocab. = Vocabulary; Inform. = Information; Word Reas. = Word Reasoning; Compr. Instr. = Comprehension of Instructions subtest from the Nepsy-II; BNT = Boston Naming Test; RDLS = Reynell Developmental Language Scales III; Expr. = Expressive Scale; Compr. = Comprehension Scale; EOWPVT = Expressive One Word Picture Vocabulary Test; Pict. Naming = Picture Naming; Receptive Voc. = Receptive Vocabulary; ROWPVT = Receptive One Word Picture Vocabulary Test; Sentence Repet. = Sentence Repetition subtest from the Nepsy-II; Forward Mem. = Forward Memory subtest from Leiter International Performance Scale - Revised; Std. Score = Standard score.

[a] Wechsler Preschool and Primary Scale of Intelligence, Third edition (Wechsler, 2009). [b] Nonverbal reasoning score is the mean of sample standardized z scores of Matrix reasoning and Block design raw scores.

* p < .05, ** p < .01, *** p < .001 for the null hypothesis ρ = 0.
